# PERM1 regulates energy metabolism in the heart *via* ERRα/PGC−1α axis

**DOI:** 10.3389/fcvm.2022.1033457

**Published:** 2022-11-07

**Authors:** Shin-ichi Oka, Karthi Sreedevi, Thirupura S. Shankar, Shreya Yedla, Sumaita Arowa, Amina James, Kathryn G. Stone, Katia Olmos, Amira D. Sabry, Amanda Horiuchi, Keiko M. Cawley, Sean A. O’very, Mingming Tong, Jaemin Byun, Xiaoyong Xu, Sanchita Kashyap, Youssef Mourad, Omair Vehra, Dallen Calder, Ty Lunde, Tong Liu, Hong Li, J. Alan Mashchek, James Cox, Yukio Saijoh, Stavros G. Drakos, Junco S. Warren

**Affiliations:** ^1^Department of Cell Biology and Molecular Medicine, Rutgers New Jersey Medical School, Newark, NJ, United States; ^2^Fralin Biomedical Research Institute at Virginia Tech Carilion, Virginia Tech, Roanoke, VA, United States; ^3^Nora Eccles Harrison Cardiovascular Research and Training Institute, University of Utah, Salt Lake City, UT, United States; ^4^Department of Microbiology, Biochemistry, and Molecular Genetics, Center for Advanced Proteomics Research, Rutgers New Jersey Medical School and Cancer Institute of New Jersey, Newark, NJ, United States; ^5^Metabolomics Core Research Facility, University of Utah, Salt Lake City, UT, United States; ^6^Department of Biochemistry, University of Utah, Salt Lake City, UT, United States; ^7^Division of Cardiovascular Medicine, University of Utah School of Medicine, Salt Lake City, UT, United States; ^8^Center for Vascular and Heart Research, Fralin Biomedical Research Institute at Virginia Tech Carilion, Virginia Tech, Roanoke, VA, United States; ^9^Department of Human Nutrition, Food and Exercise, Virginia Tech, Blacksburg, VA, United States; ^10^Division of Developmental Genetics, Institute of Resource Developmental and Analysis, Kumamoto University, Kumamoto, Japan

**Keywords:** PERM1, heart, ERRα, metabolomics, proteomics, metabolism, transcription control

## Abstract

**Aims:**

PERM1 is a striated muscle-specific regulator of mitochondrial bioenergetics. We previously demonstrated that PERM1 is downregulated in the failing heart and that PERM1 positively regulates metabolic genes known as targets of the transcription factor ERRα and its coactivator PGC-1α in cultured cardiomyocytes. The aims of this study were to determine the effect of loss of PERM1 on cardiac function and energetics using newly generated *Perm1*-knockout (*Perm1*^–/–^) mice and to investigate the molecular mechanisms of its transcriptional control.

**Methods and results:**

Echocardiography showed that ejection fraction and fractional shortening were lower in *Perm1*^–/–^ mice than in wild-type mice (both *p* < 0.05), and the phosphocreatine-to-ATP ratio was decreased in *Perm1*^–/–^ hearts (*p* < 0.05), indicating reduced contractile function and energy reserves of the heart. Integrated proteomic and metabolomic analyses revealed downregulation of oxidative phosphorylation and upregulation of glycolysis and polyol pathways in *Perm1*^–/–^ hearts. To examine whether PERM1 regulates energy metabolism through ERRα, we performed co-immunoprecipitation assays, which showed that PERM1 bound to ERRα in cardiomyocytes and the mouse heart. DNA binding and reporter gene assays showed that PERM1 was localized to and activated the ERR target promoters partially through ERRα. Mass spectrometry-based screening in cardiomyocytes identified BAG6 and KANK2 as potential PERM1’s binding partners in transcriptional regulation. Mammalian one-hybrid assay, in which PERM1 was fused to *Gal4* DNA binding domain, showed that the recruitment of PERM1 to a gene promoter was sufficient to activate transcription, which was blunted by silencing of either PGC-1α, BAG6, or KANK2.

**Conclusion:**

This study demonstrates that PERM1 is an essential regulator of cardiac energetics and function and that PERM1 is a novel transcriptional coactivator in the ERRα/PGC-1α axis that functionally interacts with BAG6 and KANK2.

## Introduction

The heart is the muscle which never rests and consumes more energy than any other organ in the body. The healthy human heart hydrolyses greater than 6 kg of ATP per day, which is mainly utilized by myosin ATPase to generate force for contractility (1). Aberrations in energy production occur in chronic heart failure. Metabolic remodeling in the failing heart includes downregulation of fatty acid metabolism and mitochondrial dysfunction, which further aggravates cardiac contractile dysfunction in the failing heart (2).

Energy metabolism is controlled and fine-tuned by a complex plethora of transcription factors and their coactivators targeting expression of metabolic enzymes and transporters (3). Prominent among those is the Estrogen-Related Receptor Alpha (ERRα), a key transcription factor that coordinates the expression of genes related to oxidative phosphorylation, fatty acid oxidation, and muscle contraction in the heart (4). ERRα belongs to ERR subfamily that includes three isoforms: ERRα (*Esrra*), β (*Esrrb*), and γ (*Esrrg*). ERR is known as an “orphan” nuclear receptor, having no known ligands and requiring the interaction with co-activators for transactivation (5). ERRα and ERRγ are highly expressed in tissues with high energy needs including the heart. Both ERRα and ERRγ bind to an ERR-alpha response element (ERRE) containing a single consensus half-site, 5′-TNAAGGTCA-3′. ERRα and ERRγ are partly redundant in their regulatory function (6), but may play distinct roles in response to pathological stress in the heart (4).

The peroxisome proliferator-activated regulator gamma coactivator-1 (PGC-1) family of transcriptional coactivators plays a critical role in the regulation of mitochondrial biogenesis and energy metabolism in the heart (7, 8). Two members of this family, PGC-1α and PGC-1β, are highly expressed in cardiac muscle and play mostly redundant roles as co-activators of ERRs (9). There is large body of evidence supporting functional co-dependency between PGC-1 coactivators and ERR family, which have led to a notion of “ERR/PGC-1 axis” of metabolic regulation in a variety of biological contexts (3, 10).

The past few years have witnessed an interesting refinement to the concept of “ERR/PGC-1 axis” in the form of organ- or tissue- specific modulators of this regulatory network (11, 12). In particular, we found that the histone methyltransferase SMYD1, expressed predominantly in skeletal and cardiac muscle, positively regulates the expression of PGC-1α (13) as well as that of PERM1 (PGC- 1-, ERR-induced regulator, muscle 1) (14). PERM1 was initially identified as a new downstream target of PGC-1 and ERRs that regulates mitochondrial bioenergetics in skeletal muscle through an undefined mechanism (15). Similar to SMYD1, PERM1 is predominantly expressed in cardiac and skeletal muscle (15). Thus, it seems that both SMYD1 and PERM1 modulate the ERR/PGC-1 regulatory axis to meet the unique energy demands in organs translating energy to mechanical work. Downregulation of the ERR/PGC-1 regulatory axis is an important component of the pathobiology of heart failure (3). As the first indication of the relevance of PERM1 to this highly prevalent disease, we have showed that PERM1 is downregulated in the human and mouse failing hearts, and that silencing of PERM1 in cultured cardiomyocyte leads to reduced mitochondrial respiration capacity (14).

Here we embarked on a comprehensive analysis of the specific role of PERM1 in regulation of cardiac metabolism, and the molecular mechanism of this regulation. The results presented here reveal broad and complex influence of PERM1 on substrate metabolism which appears to be essential for maintenance of full pumping capacity of the heart. Furthermore, we demonstrate that PERM1 acts as a novel transcriptional coactivator forming transcriptional complex with ERRα and PGC-1α, as well as transcription regulators BAG6 and KANK2 revealed as PERM1 binding partners through unbiased mass-spectrometry analysis (12).

## Materials and methods

### Ethical statements

All animal experiments were approved by the University of Utah and the Rutgers New Jersey Medical School Institutional Animal Care and Use Committee and conducted according to the Guide for the Care and Use of Laboratory Animals (The National Academy Press (16) 8th edition, 2010).

### Animals and tissue harvest

All animal experiments were approved by the University of Utah and the Rutgers New Jersey Medical School Institutional Animal Care and Use Committee and conducted according to the Guide for the Care and Use of Laboratory Animals (The National Academy Press (16) 8th edition, 2011). Perm1-KO mice were generated by Crispr/Cas9 system with gRNA targeting at the exon 2 in Sadoshima lab at the Rutgers New Jersey Medical School. The Perm1-KO allele lacks 1,157 bp in exon 2 and induces frame shift and an artificial stop codon. Genotyping of WT and mutant mice was performed using the primers, which target the Crispr target region (see Supplementary Methods for primer sequence). The heterozygous and homozygous mice and their littermates at the age of 10–12 weeks were used in this study. Echocardiography was performed on a Vevo 2100 (Visual Sonics) to determine cardiac parameters in live mice as previously described (14). Tissue samples from ventricles were immediately frozen in liquid nitrogen and stored at −80°C until they were used for analysis.

### Echocardiographic analysis

The heterozygous and homozygous mice and their littermates at the age of 9–15 weeks were used in this study. Mice were anesthetized (2% isoflurane mixed with 0.5 L/min 100% O_2_) in the induction chamber prior to the imaging studies. All echocardiographic analysis of those mice were performed using a Vivo 2100 (Visual Sonics) at the University of Utah as previously described (17).

### Surgery

Constriction of the transverse thoracic aorta (TAC) was performed on 3-month-old WT male mice at the Rutgers New Jersey Medical School, as previously described (14, 18). Thirty minutes before surgery a single dose of ZooPharm Buprenorphine SR (sustained release) (0.15 mg/kg; 72 h efficacy) was administered subcutaneously. Mice were, then, anesthetized with isoflurane (2% in O_2_) through nosecone, which was delivered from a VetFlo Vaporizer. Lidocaine (7 mg/kg) was introduced under the skin at the incision site. During recovery, if the animal was showing signs of distress, buprenorphine (regular release) was administered at 8-h intervals for up to 48 h as needed. In addition to buprenorphine SR, the NSAID carprofen (5 mg/kg) was given subcutaneously after induction of anesthesia and redosed once every 24 h for 72 h post-surgery. Mice were sacrificed 1 week after TAC surgery to collect cardiac tissue. Unbanded WT mice (sham) were used as controls.

### Histology

Whole hearts were rapidly excised from WT and Perm1-KO mice at the age of 10 weeks and were fixed in 4% paraformaldehyde and then embedded in paraffin. Hearts were then sectioned at 4 μm and placed on slides. Masson’s trichrome (Sigma) staining was performed according to the manufacturer’s protocols. Tissue sections were visualized with imaged on Nikon eclipse TE2000-U microscope with SPOT software (Diagnostic Instruments).

### Primary cultures of neonatal rat ventricular myocytes

Primary cultures of ventricular cardiac myocytes were prepared from 1-day-Crl:(WI) BR-Wistar rats (Harlan) as previously described in our publication (14). Briefly, a cardiac myocyte- and fibroblast-rich fraction was obtained by centrifugation through a discontinuous Percoll gradient. Cells were cultured in complete medium containing Dulbecco’s modified Eagles’s medium/F12 supplemented with 5% horse serum, 4 μg/ml transferrin, 0.7 ng/ml sodium selenite, 2 g/l bovine serum albumin (fraction V), 3 mM pyruvate, 15 mM Hepes pH 7.1, 100 μM ascorbate, 100 mg/l ampicillin, 5 mg/l linoleic acid, and 100 μM 5-bromo-2′-deoxyuridine (Sigma). Culture dish were coated with 0.3% gelatin. For silencing of genes, small interfering RNA (siRNA) were used, which were obtained from Qiagen.

### Plasmids

Mammalian expression vector for *Perm1* (pDC316-*Perm1*) and luciferase reporter gene driven by 3 repeats of ERRE (3xERRE-luc) were described previously (14). C-terminal Flag tagged PERM1 were generated with pDC316 (pDC316-*Perm1*-Flag). *Gal4* DNA binding domain fused *Perm1* (pFA-*Perm1*) was generated with insertion of *Perm1* cDNA into pFA-CMV (Agilent).

### Adenovirus vectors

Adenoviruses harboring *Perm1*-Flag were made using the AdMax system (Microbix) with the shuttle vectors pDC316- *Perm1*-Flag.

### High-energy phosphates profiling

ATP, ADP, AMP, PCr, creatine, and NAD^+^ levels were determined using Shimadzu HPLC system as described in our previous work (19, 20). The HPLC system was composed of CMB-20Lite, LC-20D HPLC pump, micro mixer MR100, SPD-20 UV detector, DGU-403 3-channel degasser, and RT-7725I manual injector with a fixed loop of a 20 μl capability. For the preparation of samples, approximately 10 mg of frozen tissue was homogenized in 120 μl of 0.4 M perchloric acid using a ceramic bead tube kit (MO BIO Laboratories, Inc., Carlsbad, CA, USA) and Bead Ruptor (Omini International, Kennesaw, GA, USA), which provides ultra-rapid shaking. After precipitation with 1 M KOH, the extracts were centrifuged for 3 min at 4°C two times (13,000 × *g*) Twenty microliters of the extracted sample was manually injected to the Shimadzu HPLC system.

The chromatographic separation of high-energy phosphates and relative metabolites were performed using a C18 reverse-phase column YMC-Pack Pro C18 (5 μm, 120 Å, 150 mm × 4.6 mm I.D.). The mobile phase was composed of 215 mM potassium dihydrogen phosphate, 2.3 mM TBAHS, 4% acetonitrile, 0.4% potassium hydroxide (1M), and the flow rate was set at 1 ml/min. The sample injection volume was 20 μl and the components were monitored at 210 nm (for creatine and PCr) and 254 nm (adenine nucleotides) during isocratic acquisition. All instruments and the columns were operated at room temperature (22–25°C). Standard stock solutions were freshly prepared on the day of experiments (ATP, ADP, AMP, PCr, creatine, and NAD^+^, all from Sigma, St Louis, MO, USA), and dilution was made by adding mobile phase immediately before each assay, which was used as references for peaks quantification. The cocktail of these standards was run before and after actual samples to make sure that the system performance did not decline.

### Proteomic analysis of whole lysate tissue

Proteomic analysis of ventricular tissue collected from *Perm1*^–/–^ mice and WT littermates at the age of 10 weeks were conducted using label-free quantitative LC-MS/MS, as described in our previous publication (21), with some modifications.

### Metabolomic analysis

Ventricular samples from WT and *Perm1*^–/–^ hearts were analyzed using two metabolomic platforms [GC-TOF MS and hydrophilic interaction chromatography (HILIC)-quad-time of flight (QTOF)/MS] for untargeted metabolomic screening, as previously performed (13).

### Lipidomic analysis

Agilent 6490 triple quadrupole (QqQ) mass spectrometry was used to perform lipidomic profiling of WT and *Perm1*^–/–^ hearts, as described in our previous publication (22).

### Bioinformatic analysis of -omics data

Principal component analysis (PCA), and the analyses of heart maps, fold-change, and volcano plots were carried out using the online software Metaboanalyst (23). Enrichment analyses and the generation of network maps of proteome were carried out using the online software STRING database.

### Mass-spectrometry-based screening of PERM1-bound proteins in cardiomyocytes

*Perm1*-Flag was expressed in primary cultured cardiomyocytes with adenovirus vector (10 cm dish), followed by immunoprecipitation with anti-Flag antibody (Sigma, A2220). The immune-complex was subjected to SDS-PAGE followed by mass spectrometric analysis.

### Chromatin immunoprecipitation assay

Heart tissue was cross-linked with formaldehyde. The nuclear fraction was isolated and sonicated to generate a chromatin solution that was then used for immunoprecipitation. Primers used for investigating mouse genome are as follows: (5′ to 3′). Cpt2–CCCTGTGGGCGGAGTTGAACT and ATTTTGTCCGTGACCTTCGCGC; Mcad–ATCTAGCCCAGAATTTGTTGTTCCAGTG and GCGGTGGCTGAGGGAGTTCC; Idh3a–TGATGGATGGCGGAGGCGAGC and TGGGGACACCCGGAGCAGTAC; Sdhd–CCTTCCGGTTCACGCTTCAGGT and TCGCTTTCGAGGGCTCAAGGT; ERRα–CTCGTGTCTCACCTCTGCCTTT and GACACTTGACAGATTTGGTAGATCAG; Ndufv1–GCTCCCCACCCCGTGTGTTT and GGGAGCCAGACGGGTCACCT; and Ndufs1–CTTATATAGAGACGGAGGCGTTTC and GACGACCTGTGGGCCCTTTAGTTA. The corrected chromatin fragment was validated by qPCR with Maxima SYBR Green qPCR master mix (Fermentas).

### *In vitro* DNA binding assay

DNA binding assay using the *Idh3a* promoter was performed in cultured neonatal rat cardiomyocytes, as described in our previous publication (18) with some modifications. Briefly, biotin-labeled *Idh3a* promoter (380 bp) was prepared by PCR with a biotin-labeled primer. Endogenous ERRα was knocked down in primary cultured cardiomyocytes by siRNA transfection. After 2–3 days of transfection, the cells were lysed with 300 μl of a nuclear lysis buffer (25 mM Hepes, pH7.9, 0.1 mM EDTA, 10 mM Sodium Butyrate, 10 mM NaF, 1 mM DTT, 1% NP-40, 420 mM KCl, 1 × Proteinase inhibitor cocktail). The 100 μl lysate was diluted with 300 μl of a dilution buffer (10 mM Hepes, pH 7.9, 2.5 mM MgCl2, 5% Glycerol, 1 mM DTT, 0.1% NP-40). The total 400 μl of diluted lysate was incubated with 1 μg biotin-labeled DNA (*Idh3a* promoter) and streptavidin-beads at 4°C for 2–4 h with rotation. The protein-DNA complex was washed with 1 ml of a washing buffer (10 mM Hepes, pH 7.9, 2.5 mM MgCl 2, 5% Glycerol, and 50 mM KCl 1 mM DTT, 0.1% NP-40) for 3 times. The protein bound to the DNA was analyzed with Western blot analyses.

### Luciferase reporter gene assay

Luciferase assays were performed in primary cultured rat cardiac myocytes as previously performed (13). Briefly, reporter plasmids (0.3 μg per well), including 3xERRE-luc and UAS-luc, mammalian expression vectors (0.7 μg per well), including pDC316 control vector, pDC316-Perm1 and pFA-Perm1, and siRNA (20 pM) against PGC-1α, ERRα, Ankrd1, Bag6, Kank2, and TIF1β, were transfected into cells plated in 12 well plates using LipofectAmine 2000 (Invitrogen). Total plasmids were kept at 1 μg per well using pDC316 control vector. The total siRNA was kept 20 pM per well using control siRNA. Two μl LipofectAmine 2000 was used per well. Fifty μl OPTI-MEM was used for dilution of plasmid vectors and of LipofectAmine 2000, respectively. Luciferase assays were performed 2 days after transduction with a luciferase assay system (Promega).

### Transmission electron microscopy

Conventional electron microscopic analyses were performed with heart tissue from WT and *Perm1*^–/–^ mice. Morphometric analysis of mitochondrial cristae and quantification of density and disorganized mitochondrial cristae were performed as previously demonstrated (24).

### Immunoblotting

Total protein lysates (10–30 μg) from mouse left ventricle tissue were incubated with SDS sample buffer at 95°C for 5–20 min. These denatured protein samples were transferred to either polyvinylidene difluoride or nitrocellulose membranes and probed with antibodies against Perm1 (Sigma HPA032711), ERRα (Millipore ERR46Y), PGC-1α (Millipore Ab3242), Ankrd1 (Novus NBP2-15397), BAG6 (Cell Signaling Technology 8523), Kank2 (Novus NBP3-04645), TIF1b (Cell Signaling Technology 4123), and Trx1 (Cell Signaling Technology 2429). The complete methods are in Supplementary material online.

### Immunoprecipitation

PERM1 and ERRα were immunoprecipitated from mouse heart lysate with anti-PERM1 (Sigma HPA032711) and anti-ERRα antibody (PPMX PP-H5844-00). Protein A/G agarose (Santa Cruz sc-2003) was used.

### Gene expression analysis

Real-time PCR was performed with either Taqman method or the SYBR green method on a CFX96 Real-Time PCR system (Bio-Rad) as described previously (13, 22). Briefly, RNA was extracted using TRizol (Life Technologies), followed by ethanol extraction. Reverse transcription was performed from 1 μg of RNA using QuantiTect Reverse Transcription Kit according to the manufacture’s instruction (Qiagen). Relative expression was calculated using the ΔΔCt method with normalization to either *Tbp* or β-actin. The complete methods and a list of primers that were used in this study are in Supplementary material online.

### Statistical analysis

For comparisons between WT, *Perm1*^±^ and *Perm1*^–/–^, one-way ANOVA with Fisher’s LSD *post-hoc* test was applied using GraphPad Prism version 9. Student’s *T*-test was used to compare WT vs. *Perm1*^–/–^ mice. A value of *p* < 0.05 was considered statistically significant. Data are given as mean ± SEM.

## Results

### *Perm1*-knockout mice exhibit reduced cardiac function

**FIGURE 1:**
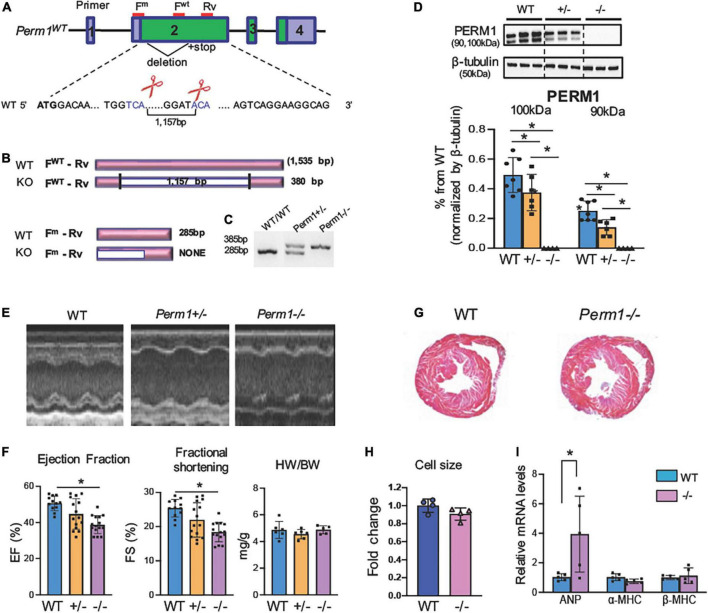
**(A)** *Perm1*^–/–^ (KO) mice exhibit reduced cardiac function (age, 3 months). (Schematic of *Perm1* gene and the CRISPR/Cas9-mediated gene editing in *Perm1* loci in C57BL/6 mice. Exon 2 was targeted, where the primers for genotyping are indicated in red lines (F^WT^ + Rv for WT; F^m^ + Rv for mutant). **(B)** Schematic of PCR products from WT and CRISPR/Cas9 mice (KO). PCR with the primer set, F^WT^ + Rv, distinguishes WT (not detected) from either *Perm1*^±^ or *Perm1*^–/–^ (360-bp band), while PCR with the primer set, F^m^ + Rv, distinguishes WT (285 bp) from Perm1^±^ or *Perm1*^–/–^ mice (not detected). **(C)** Genotyping of WT, *Perm1*^±^ and *Perm1*^–/–^ mice using those primers. **(D)** Western blotting analysis showing the reduced endogenous levels of PERM1 in *Perm1*^±^ and *Perm1*^–/–^ hearts (*n* = 6/group). **(E)** Sample 2D echocardiographs from WT, *Perm1*^±^ and *Perm1*^–/–^ mice. **(F)** Ejection fraction (EF), fraction shortening (FS), and the heart weight (mg)–body weight (g) ratios (HW/BW) of WT, *Perm1*^±^ and *Perm1*^–/–^ mice (*n* = 15/group). **(G)** Low-magnification views of hematoxylin/eosin stains of transverse sections of WT and *Perm1*^–/–^ hearts. **(H)** Quantification of cell size in WT and *Perm1*^–/–^ hearts (*n* = 5/group). **(I)** qPCR of ANF, α-MHC, and β-MH in WT and *Perm1*^–/–^ hearts (*n* = 5/group). *p < 0.05.

Our previous study showed that PERM1 is downregulated in the human and the mouse failing heart (14). To determine whether downregulation of PERM1 can be associated with cardiac dysfunction, we generated mouse strains deficient in *Perm1* (*Perm1*^–/–^ mice) using the Crispr/Cas9 genome editing technique. We targeted exon 2 to delete 1,157 bp (Figure 1A), leading to a large deletion after amino acid 27 in the *Perm1* locus (Figure 1B). *Perm1*^±^ and *Perm1*^–/–^ mice were obtained from offspring of heterozygous breeding pairs. Genotypes of heterozygotes and homozygotes were confirmed by hybridization and PCR analysis of genomic DNA (Figure 1C). Consistent with previous studies, we detected two isoforms of PERM1 at 100 kDa and 90 kDa, both of which significantly decreased in *Perm1*^±^ hearts (44.3 and 20.2% of WT in 100 and 90 kDa, respectively, both *p* < 0.05, Figure 1D). PERM1 was undetectable in *Perm1*^–/–^ hearts (Figure 1D). Pups lacking *Perm1* were born at the expected Mendelian ratio (20.3, 54.2, and 25.4% in WT, *Perm1*^±^ and *Perm1*^–/–^ mice), suggesting that PERM1 is dispensable for embryonic development. Echocardiographic analysis revealed decreased cardiac function in *Perm1*^–/–^ mice. Ejection fraction (EF) and fractional shortening (FS) were reduced in *Perm1*^±^ and *Perm1*^–/–^ mice as compared with WT littermates, proportionally to the protein levels of PERM1 in the heart (EF: 50.1 ± 1.9 vs. 42.8 ± 2.86 vs. 34.7 ± 1.38%, FS: 24.8 ± 1.18 vs. 20.7 ± 1.76 vs. 16.0 ± 0.89% in WT, *Perm1*^±^ and *Perm1*^–/–^, respectively; Figures 1E,F and Supplementary Table 1, *n* = 15 per group). The differences in EF and FS were significant between *Perm1*^–/–^ and WT mice (both *p* < 0.05), but not between *Perm1*^±^ and WT mice. There were no differences in heart-to-body weight ratios, (right panel in Figure 1F), cell size (Figure 1H), and gross-anatomy of the heart (Figure 1G) between *Perm1*^–/–^ and WT mice. Interestingly, *Perm1*^–/–^ mice exhibited a significant increase in the mRNA level of atrial natriuretic factor (ANF) (Figure 1I), which is known to be upregulated in the failing heart (25), but the related biomarkers α-MHC and β-MHC were unchanged.. In summary, our data suggest that the expression level of PERM1 in the heart is correlated to cardiac function. Thus, PERM1 is essential for maintaining the normal cardiac function.

### Loss of PERM1 reduces myocardial energy reserve

**FIGURE 2:**
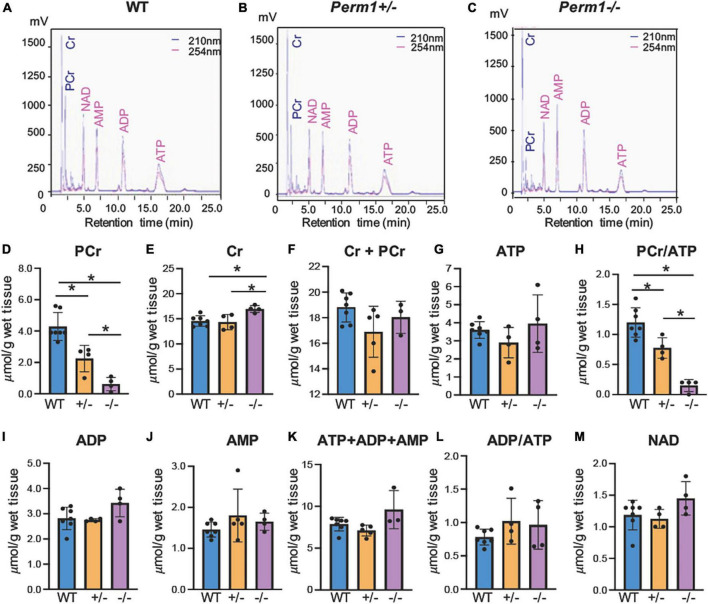
Quantification of high-energy phosphates and related adenine nucleotides. Metabolites were measured by HPLC in ventricular tissue from WT, *Perm1*^±^ and *Perm1*^–/–^ mice. Chromatograms of extracts from WT **(A)**, *Perm1*^±^ **(B)** and *Perm1*^–/–^ hearts **(C)**. **(D–M)** Quantitative analysis of the levels of creatine (Cr), phosphocreatine (PCr), ATP, ADP, AMP, and NAD^+^, respectively, normalized by wet tissue weight. *p < 0.05.

We assessed the levels of high-energy phosphates in myocardium using HPLC (Figure 2). The upper three panels (A–C) show the representative chromatograms in WT, *Perm1*^±^ and *Perm1*^–/–^ mice, respectively. The levels of phosphocreatine (PCr) were significantly decreased in *Perm1*^±^ and *Perm1*^–/–^ hearts (*p* < 0.05 in all comparison, Figure 2D). The creatine (Cr) content in *Perm1*^–/–^ hearts was significantly increased as compared to WT hearts, but there was no change in *Perm1*^±^ hearts (Figure 2E). The total creatine pool (Cr + PCr) was not statistically different among WT, *Perm1*^±^ and *Perm1*^–/–^ hearts (Figure 2F). There was no significant difference in the levels of ATP, ADP, AMP, total adenine nucleotide pool (ATP + ADP + AMP, Figure 2K), and the ADP/ATP ratio among the three groups (Figure 2G). There was a trend that the NAD level was increased in *Perm1*^–/–^ heart (*p* = 0.058, Figure 2M). Importantly, the PCr/ATP ratio, a sensitive indicator of the energy state, was significantly decreased in both *Perm1*^±^ and *Perm1*^–/–^ hearts (*p* < 0.05 in all comparisons, Figure 2H). Thus, like cardiac function, the myocardial energy reserve was decreased proportionally to the expression levels of PERM1 in the heart.

### Global downregulation of oxidative phosphorylation proteins and upregulation of glycolysis enzymes in *Perm1*^–/–^ hearts

**FIGURE 3:**
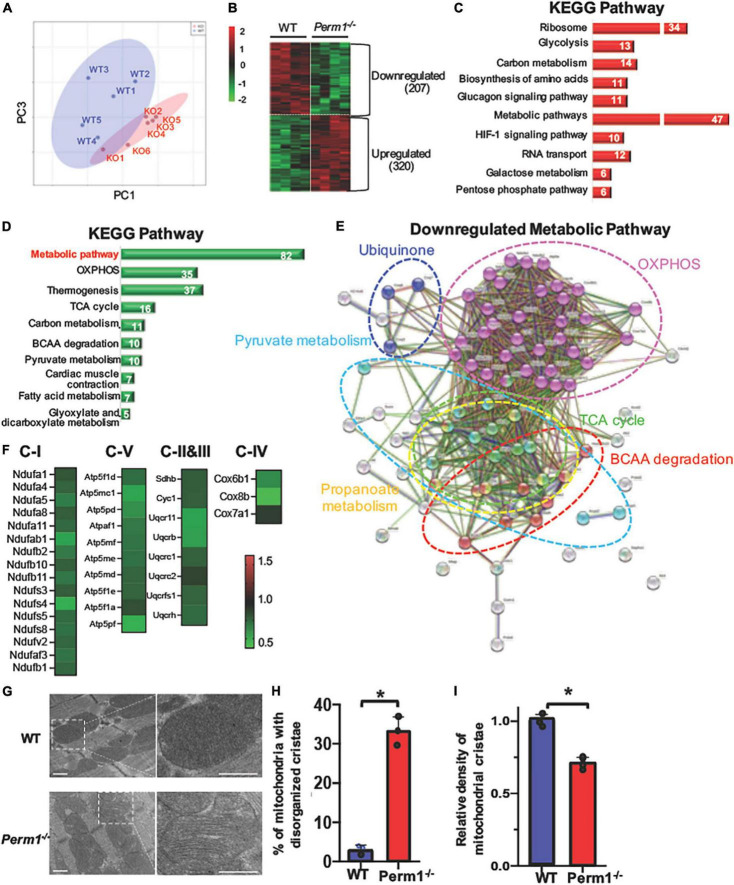
Proteomic analysis of left ventricle tissue from *Perm1*^–/–^ mice and WT mice (*n* = 5/group). Among 2,868 detected proteins, 207 proteins were significantly downregulated and 320 proteins were significantly upregulated in *Perm1*^–/–^ hearts (*p* < 0.05 by *t*-test). **(A)** PCA of all detected proteins from *Perm1*^–/–^ (*red*) and WT hearts (*blue*). **(B)** Heat map of the proteins that were differentially expressed in *Perm1*^–/–^ heart. Enrichment analyses of upregulate **(C)** and downregulated proteins **(D)** for KEGG pathways. The white numbers in bar graphs indicate the number of proteins that were detected in each pathway. **(E)** Network map of the 82 significantly changed proteins which belong to the collective KEGG term metabolic pathways in panel **(D)**. **(F)** Heat maps of fold changes from the 35 proteins comprising the network map presented in panel **(E)**. Analysis of cardiac ultrastructure with representative electron microscopy images **(G)**, quantification of changes in mitochondrial cristae morphology **(H)**, and quantification of mitochondrial cristae density **(I)** of hearts of WT mice (blue bars, *n* = 3) and *Perm1*^–/–^ mice (red bars, *n* = 3). *p < 0.05 (student’s *t*-test), ± SED.

To investigate biological pathways regulated by PERM1 in the heart, we performed LC-MS/MS-based proteomic analysis of WT and *Perm1*^–/–^ hearts (*n* = 4 per group). The PCA of the total of 2,868 detected proteins (Supplementary Dataset 1) separated WT and *Perm1*^–/–^ groups (Figure 3A), suggesting altered proteomic profile of *Perm1*^–/–^ hearts. A heat map shows 527 proteins that are differentially expressed in *Perm1*^–/–^ hearts (207 downregulated, 320 upregulated, *p* < 0.05, Figure 3B). Using the STRING Database web-based tools (26), the 10 most significant enriched Kyoto Encyclopedia of Genes and Genomes (KEGG) pathways are shown in Figures 3C,D (enrichment of upregulated proteins in Figure 3C, enrichment of downregulated proteins in Figure 3D). We found that the most upregulated metabolic pathways were annotated into the ribosome and glycolysis pathways (Figure 3C, Supplementary Dataset 2). Gene ontology (GO) terms for cellular component showed that many of upregulated proteins *Perm1*^–/–^ hearts were localized in the cytoplasm (Supplementary Figure 1A and Supplementary Dataset 3). Of note, it has been reported that ribosomal protein expression is increased in response to oxidative stress (27), while upregulation of glycolysis occurs in the progression of heart failure, concomitant with downregulation of OXPHOS (28). The two most enriched KEGG pathways of downregulated proteins in *Perm1*^–/–^ hearts are the wide, encompassing pathway terms of metabolic pathways and OXPHOS (Figure 3D, Supplementary Dataset 4), and at the top of the list for cellular component terms of downregulated proteins is mitochondria (Supplementary Figure 1B and Supplementary Dataset 5). To get the insights into the downregulation of metabolic pathway in the enrichment analysis, the interaction network view of the 82 significantly altered proteins within the metabolic pathways KEGG term was generated (Figure 3E). Note that highly interconnected clusters include proteins involved in OXPHOS (pink), TCA cycle (green), pyruvate metabolism (light blue), branched-chain amino acid (BCAA) metabolism (red), ubiquinone (blue), and propanoate metabolism (yellow). The comparative analysis of the 35 downregulated OXPHOS proteins in the enrichment analysis of downregulated proteins (Figure 3D) is depicted in heat maps (Figure 3F), displaying the protein names and fold changes.

Electron microscopic analysis of PERM1-null hearts showed that the density of mitochondrial cristae was remarkably reduced, concurrent with increased disorganization as compared to WT hearts (Figures 3G–I). Overall, these results suggest that PERM1 deletion leads to downregulation of OXPHOS proteins, upregulation of glycolysis, and altered mitochondrial morphology, all of which resembles metabolic remodeling in the failing heart (8, 29).

### Accumulation of glycolytic and polyol intermediates and the decreased contents of fatty acid and triglyceride in *Perm1*^–/–^ hearts

**FIGURE 4:**
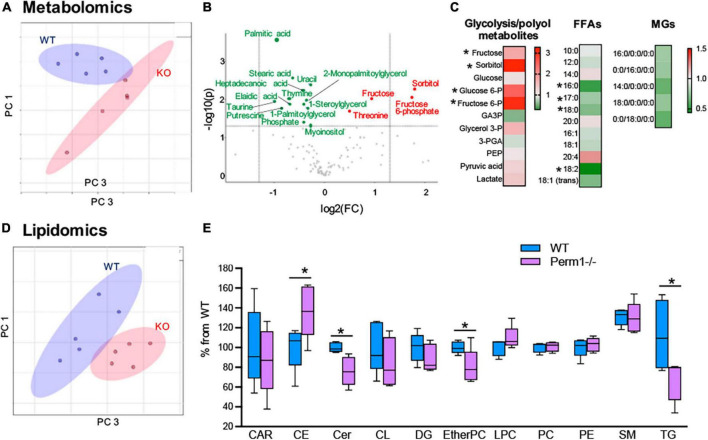
Metabolomic and lipidomic analyses of left ventricle tissue from *Perm1*^–/–^ mice and WT mice (*n* = 6/group in metabolomics and *n* = 5/group in lipidomics). **(A–C)** Metabolomic profile of *Perm1*^–/–^ hearts. **(A)** PCA of all detected metabolites in *Perm1*-null hearts (red) and WT hearts (blue). **(B)** Volcano plot contains the significantly increased (red) and decreased (green) metabolites in *Perm1*^–/–^ hearts compared with those in WT hearts (*p* < 0.05). *X*-axis indicates fold change (FC) in log2 scale and *y*-axis indicates *p*-value in -log10 scale. **(C)** Heat maps of metabolites in glycolysis and polyol pathways, and free fatty acids (FFAs) and monoglycerides (MGs). **(D,E)** Lipidomic profile of *Perm1*^–/–^ hearts. PCA of all detected lipids in *Perm1*^–/–^ (red) and WT hearts (blue) **(D)**, and box and violin plot of the total lipid species in each lipid group **(E)**. *p < 0.05.

To examine if the changes in protein expression (Figure 3) alter myocardial metabolic contents, we performed GCMS-based metabolomics and LC/MS-based lipidomics in the hearts from WT and *Perm1*^–/–^ mice. Metabolomics (*n* = 6 per group) detected 115 metabolites (Supplementary Dataset 6). The PCA plot of metabolome clearly separates the WT and *Perm1*^–/–^ groups (Figure 4A). The volcano plot in Figure 4B, where the *x*-axis indicates fold changes in log-2 scale and the *y*-axis indicates *p*-values in -log10 scale, highlights the increased levels of the intermediates of glycolysis and polyol pathways (sorbitol; fructose-6-phosphate; fructose, indicated in red, all *p* < 0.05) and the decreased levels of free fatty acids (FFAs) and monoglycerides (MGs) (palmitic acid; stearic acid; elaidic acid; 1-stearoylglycerol; 1-palmitoylglycerol, indicated in green, all *p* < 0.05). Heat maps in Figure 4C summarize the changes in glucose metabolites, FFAs, and MGs in *Perm1*^–/–^ hearts. Lipidomics (*n* = 5 per group) detected 394 lipids, including carnitines (CAR), ceramides (Cer), cholesterol ester (CE), diglycerides (DG), phosphatidylcholines (PC), phosphatidylethanolamine (PE), sphingomyelin (SM), and triglycerides (TG) (Supplementary Dataset 7). Similar to metabolome, the PCA plot of these lipids clearly separated WT and *Perm1*^–/–^ hearts (Figure 4D). The detected lipids were further grouped into the abovementioned classes using the LIPID MAPS classification system (30), and the total levels of lipids in each class were analyzed using box and violin plots (Figure 4E), which revealed global decrease of TG and ceramides (Cer), and an increase of cholesterol ester (CE), in *Perm1*^–/–^ hearts as compared with WT hearts.

**FIGURE 5:**
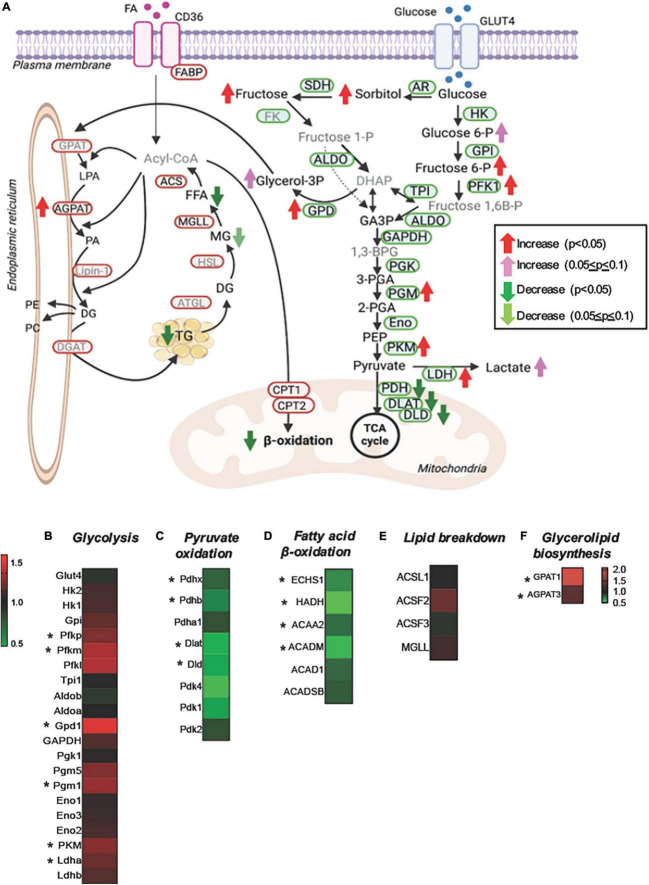
Integrated metabolomic and lipidomic analyses with proteomic data of *Perm1*^–/–^ hearts. **(A)** Schematics of glucose and lipid metabolism pathways, in which the altered levels of metabolites, lipids, and proteins are indicated by arrows. Detected metabolites and proteins are indicated in black fonts, and undetected metabolites and proteins are indicated in gray fonts (created using BioRender). Heat maps of the detected proteins involved in glycolysis **(B)**, pyruvate oxidation **(C)**, fatty acid β-oxidation **(D)**, lipid breakdown **(E)**, and glycerolipid biosynthesis **(F)**. Glucose-6-P:glucose-6-phosphate, fructose-6-P:fructose-6-phosphate, fructose-1-P:fructose-1-phosphate, fructose-1,6B-P: fructose-1,6-bisphosphate, DHAP:dihydroxyacetone phosphate, GA3P:glyceraldehyde-3-phosphate, glycerol-3P:glycerol-3-phosphate, 1,3-BPG:1,3-bisphosphoglycerate, 3-PGA:3-phosphoglycerate, 2-PGA:2-phosphoglycerate, PEP, phosphoenolpyruvate; FFA, free fatty acid; MG, monoglyceride; DG, diglyceride; LPA, lysophosphatidate; PA, phosphatidate; PE, phosphatidylethanolamine; PC, phosphatidylcholines. *p < 0.05.

For a better understanding of the changes in the substrate metabolism due to *Perm1* deletion, we created an integrative view combining all metabolomic, lipidomic, proteomic, and gene expression data yielded by this study (Figure 5). As shown in Figure 5A, the accumulation of glycolytic intermediates (glucose-6-P; fructose-6-P, and lactate) was associated with global upregulation of glycolytic enzymes (GPI; PFK1; PGM; PLM, all *p* < 0.05, Figures 5A,B). qPCR analysis showed that both GLUT1 and GLUT4 were upregulated in *Perm1*^–/–^ mice (both *p* < 0.05, Supplementary Figure 2A). Upregulation of lactate dehydrogenase (LDHA, *p* < 0.05) concomitant with downregulation of the pyruvate dehydrogenase complex (ODHB; PDHX; DLAT; DLD, Figure 5C) and the increased myocardial level of lactate (FC = 1.43, *p* = 0.088) suggest the increased reliance on anaerobic metabolism in *Perm1*^–/–^ hearts. We must note that the increased levels of sorbitol and fructose in *Perm1*^–/–^ hearts (FC = 3.28, *p* < 0.05) were not associated with a significant change in the protein expression of aldose reductase (AR, AKR1B10) and sorbitol dehydrogenase (SDH, SORD), which catalyze glucose to sorbitol and further to fructose, respectively (FC = 1.01 and 1.21, *p* = 0.171 and 0.174, respectively, Figure 5A). It is worth noting that one of the most significantly upregulated glycolytic enzymes in *Perm1*^–/–^ hearts was glycerol-3-P dehydrogenase (*GPD1*, FC = 1.414, *p* = 0.01, Figures 5A,B), which serves as a major link between glycolysis and lipid biosynthesis. The initial step of glycerolipid biosynthesis is catalyzed by glycerol-3-P acyltransferase (GPAT), which esterify fatty acid acyl-CoA (acyl-CoA) to glycerol-3-phosphate, yielding lysophosphatidic acid (LPA). Our proteomic data showed that GPAT1 was significantly upregulated in *Perm1*^–/–^ hearts (Figure 5F), concomitant with upregulation of 1-acylglycerol-3-phosphate acyltransferase (AGPAT) (Figures 5A,F), the latter catalyzing the acylation of LPA to form phosphatidic acid (PA). Taken together, these data suggest that the glycerolipid biosynthesis was promoted in *Perm1*^–/–^ hearts. However, despite the upregulation of the enzymes responsible for glycerolipid biosynthesis (GPD; GPAD; AGPAT), the myocardial levels of TGs, a major energy storage in cardiomyocytes, were reduced in *Perm1*^–/–^ hearts (Figures 4E,F, 5A), indicating the imbalanced input and output in the TG pool. We did not observe significant changes in the protein levels of enzymes involved in lipid breakdown from TGs in *Perm1*^–/–^ hearts (ACS; MGL, Figures 5A,E). Importantly, the key enzymes involved in fatty acid β-oxidation (FAO) were consistently downregulated in *Perm1*^–/–^ hearts (ACADM; ACAA2; HADH; ECHS1, Figure 5D). qPCR data showed that the mRNA levels of CD36, which facilitates fatty acid uptake into muscle cells, were significantly decreased in *Perm1*^–/–^ hearts, whereas there was no significant difference in the mRNA levels of CPT1 and CPT2, which are responsible for fatty acid uptake into mitochondria for FAO (FC = 0.84 and 0.96, respectively, both *p* > 0.05, Supplementary Figure 2B). Of note, qPCR analysis also showed downregulation of the OXPHOS genes *Idh3a* and *Ndufv1* in *Perm1*^–/–^ mice, which are consistent with our previous study in cardiomyocytes (both *p* < 0.05, Supplementary Figure 2C).

Summarizing, the unique metabolic profile induced by *Perm1* deletion combines evidence for reduced lipid utilization and storage, partially upregulated glycerolipid biosynthesis, accumulation of glycolytic and polyol intermediates, and downregulation of pyruvate oxidation.

### PERM1 interacts with ERRα in cardiomyocytes and mouse hearts

**FIGURE 6:**
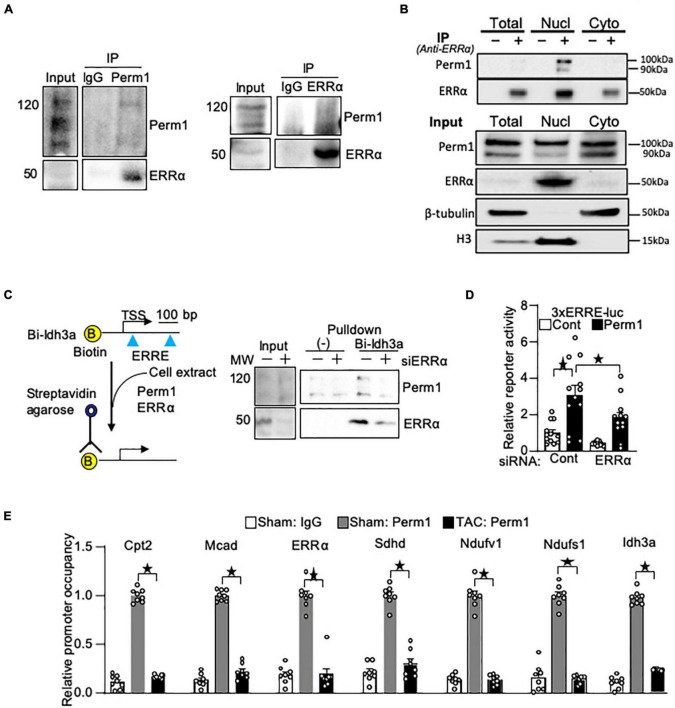
PERM1 interacts with ERRα and activates ERRE in an ERRα-dependent manner. **(A)** PERM1 binds to ERRα in the adult mouse heart. **(B)** Co-IP assay of subcellular fractionation from the mouse adult heart shows that PERM1 binds to ERRα in the nucleus. **(C)** *In vitro* DNA binding assay using biotin-labeled DNA comprising the promoter of *Idh3a* (ERR target gene) and cardiomyocyte lysate shows that PERM1 bound to the ERRE of the ERR target gene, which was partly inhibited by silencing of ERRα (siERRα). **(D)** Reporter gene assays were performed in cardiomyocytes using a luciferase reporter gene driven by 3 repeats of EER response element (3xERRE-luc) (*n* = 12/group). Adenovirus-mediated overexpression of PERM1 increased the ERRE activity (white bar vs. black bar, scrambled-siRNA), which was partially inhibited by silencing of ERRα (siERRα). Cont: control (adenovirus-null), PERM1: adenovirus-PERM1 (overexpression) **(E)** ChIP-PCR was performed with anti-PERM1 antibody in the mouse heart that was subjected to either sham or 4 weeks of transverse aortic constriction (TAC) surgery (*n* = 8/group). PERM1 was localized to the flanking promoter region (ERRE) of the well-known ERR target genes (*Ctp2*; *Mcad*; *ERRα*; *Sdhd*; *Ndufv1*; *Ndufs1*; *Idh3a*), which was abolished by pressure overload. *p < 0.05 (one-way ANOVA), ± SED.

We previously demonstrated that Perm1 activates transcription through ERR response element (ERRE) (14) where ERRα binds for the transcriptional activation of its downstream target genes. Thus, we hypothesized that PERM1 regulates energy metabolism through ERRα. To test if PERM1 physically interacts with ERRα as part of its transcription complex, we performed co-immunoprecipitation (Co-IP) assays in cardiac tissue from WT mice. ERRα was co-immunoprecipitated with PERM1 using mouse heart lysate (Figure 6A, left), whereas Perm1 was pulled down by anti-ERRα antibody (Figure 6A, right), indicating the binding of Perm1 with ERRα in the heart. Furthermore, we performed Co-IP assay using subcellular fraction of WT mouse cardiac tissue. As shown in Figure 6B, PERM1 was pulled down by anti-ERRα in the nucleus fraction, suggesting that PERM1 could be a transcription partner of ERRα in the heart. These data suggest that PERM1 forms transcriptional complex with both ERRα and PGC-1α. To test if the recruitment of PERM1 to the ERRE depends on ERRα, we performed *in vitro* DNA binding assay using biotin-labeled DNA comprising 380 bp of the promoter of *Idh3a*, an ERRα target gene, containing ERREs and a transcription start site (TSS) (Figure 6C). The promoter was incubated with cell lysate from cardiomyocytes, which were transfected either with scrambled-siRNA (control, “siERRα-“) or siRNA for ERRα (siERRα, ERR knockdown, “siERRα + “). PERM1 was recruited to the promoter of *Idh3a*, which was partly inhibited by ERRα knockdown, suggesting that PERM1 was localized to the promoter of the ERR target gene partly through ERRα. Next, we examined if PERM1 activates the transcription of the ERRE in an ERRα-dependent manner. A reporter gene assay was performed using the cardiomyocytes that were treated with either scrambled-siRNA or siERRα, followed by transfection with a construct that contains a luciferase reporter gene driven by a minimal promoter having upstream 3 repeats of ERR response element (ERRE), 3xERRE-luc. Consistent with our previous observation (14), the reporter activity of 3xERRE-luc was significantly increased by overexpression of PERM1, as compared with control vector, which was partially, yet, significantly decreased by ERRα knockdown (siERRα) (white bar vs. black bar in “ERRα,” *p* > 0.05; “scrambled” vs. “ERRα” in black bars, *p* < 0.05 in Figure 6D), suggesting that PERM1 activates the ERRE transcription, in part, through ERRα. To test whether PERM1 is localized to the ERR target gene promoters in the heart, ChIP assays were performed using ventricular tissue from WT mice that were subjected to either sham or TAC surgery, the latter of which induces pressure overload in the heart. As shown in Figure 6E, PERM1 was localized to the proximal region of ERRE that are present in the promoters of ERRα and its target genes *(Cpt2*; *Mcad*; *Sdhd*; *Ndufv1*; *Ndufs1*; *Idh3a*), while the occupancy of PERM1 on the ERRE of ERRα and those ERR target gene promoters was reduced in the TAC mouse heart, suggesting that the transcriptional regulation of ERR target genes by PERM1 was decreased during pressure overload (Figure 6E). These results are consistent with downregulation of PERM1 in the mouse TAC hearts, which we previously shown (14). Taken together, these results suggest that PERM1 positively regulates the transcription of ERR target genes by localizing to and activating the ERRE, likely through interacting with ERRα.

### PERM1-induced transcriptional activation requires specific binding partners

**FIGURE 7:**
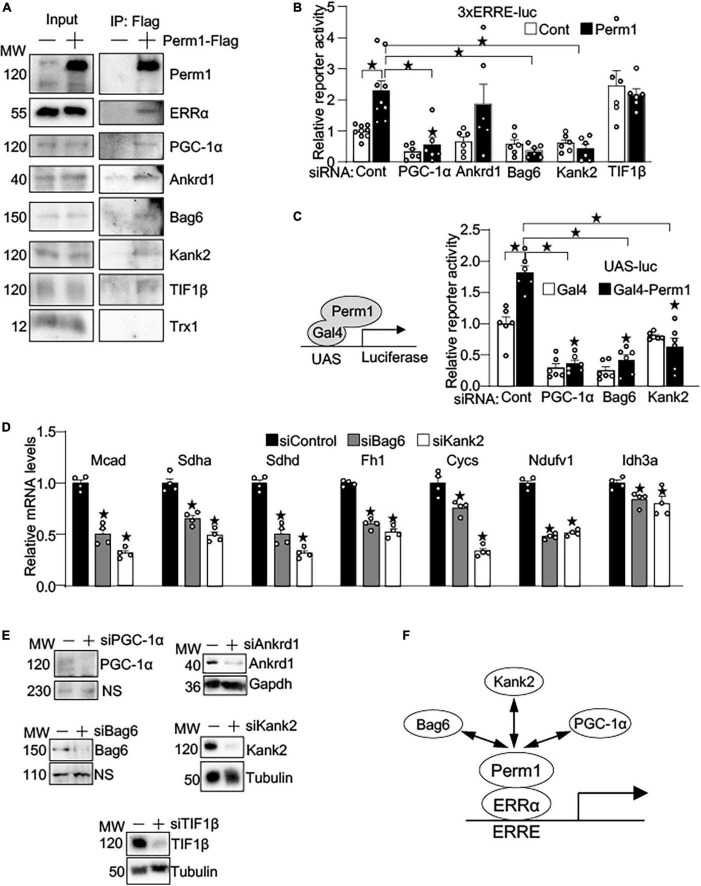
PERM1 is a novel transcription coactivator that functionally interacts with PGC-1α, BAG6, and KANK2. **(A)** Immunoprecipitation assays show that PERM1 binds to the transcriptional regulators ERRα, PGC-1α, ANKRD1, BAG6, KANK2, and TIF1β. Adenovirus Flag-tagged *Perm1* (PERM1-Flag) was transduced to cardiomyocytes, and PERM1-bound proteins were pulled down by anti-Flag antibody. Immunoblotting confirmed the interaction of PERM1 with ERRα, PGC-1α, ANKRD1, BAG6, KANK2, and TIF1β. In contrast, TRX1, which was also identified through MS-based screening as a potential binding partner in transcription regulation, did not bind to PERM1. **(B)** Luciferase reporter gene assays show that the PERM1-induced transcriptional activation of the ERRE requires PGC-1α, BAG6, and KANK2 (*n* = 7-9/group). Cardiomyocytes were transduced with 3xERRE-luc, followed by transfecting with either scrambled-siRNA (scr), siPGC-1α, siANKRD1, siBAG6, siKANK2, or TIF1β. **(C)** *In vitro* Gal4 assay shows the recruitment of PERM1 to a gene promoter induces transcriptional activation (*n* = 6/group). Gal4-fused *Perm1* (Gal4-*Perm1*) was expressed with UAS-luc, a reporter gene driven by Gal4 binding sequence, in cardiomyocytes. In control (scrambled-siRNA, “scr”), the reporter activity was significantly increased by Gal4-*Perm1*, indicating that PERM1 can act as a transcription coactivator. Silencing of PGC-1α, BAG6, KANK2 inhibited the transcription activation by PERM1. **(D)** qPCR shows downregulation of ERR target genes by silencing BAG6 and KANK2 (siBag6 and siKank2) in cultured cardiomyocytes (*n* = 4/group). **(E)** siRNA-mediated knockdown of PGC-1α, ANKRD1, BAG6, KANK2, or TIF1β were verified by western blotting analyses. **(F)** Hypothetical model of transcriptional regulation by PERM1 on the ERRE. PERM1 is localized to and activates the ERRE in ERR target genes through interacting with ERRα and the other transcriptional regulators BAG6, KANK2, and PGC-1α (*p < 0.05).

To gain the insights into the mechanism by which PERM1 promotes transcription, we performed MS-based unbiased screening of PERM1-bound proteins. Flag-tagged PERM1 was expressed in cultured cardiomyocytes, followed by immunoprecipitation with anti-Flag antibody. PERM1-bound proteins of whole lysates were identified using LC-MS/MS. The total of 545 proteins (Supplementary Dataset 8) were analyzed for GO-oriented biological process, which identified 278 pathways that were significantly recognized as biological process (false discovery rate, FDR < 0.05, Supplementary Dataset 9). Among those pathways, we focused on “regulation of gene expression” (Supplementary Dataset 9, FDR = 0.0029), which includes ankyrin repeat domain 1 (ANKRD1), ANKYRIN2, ANKYRIN3, KN motif and ankyrin repeat domains 1 (KANK1), KANK2, BLC2-associated anthanogene-6 (BAG6), transcriptional intermediary factor 1β (TIF1β) and thioredoxin 1 (TRX1). To verify their binding to PERM1, Co-IP assays were performed. Consistent with our result shown in Figures 6A,B and a previous study (31), ERRα and PGC-1α were pulled down by anti-Flag antibody in cardiomyocytes (Figure 7A). In addition, we confirmed the interaction of PERM1 with ANKRD1, BAG6, KANK2, and TIF1β, however, the binding of PERM1 with TRX1 was not observed. To test whether those transcriptional regulators are required for the transcription activation of ERRE by PERM1, luciferase reporter gene assays were performed in cardiomyocytes that were treated with either scramble-siRNA, PGC-1α, ANKRD1, BAG6, KANK2, or TIF1β. As shown in Figure 7B, PERM1-induced transcriptional activation via ERRE was inhibited by knockdown of PGC-1α, BAG6, and KANK2, while silencing of *Ankrd1* did not affect the ERRE activation by PERM1. Unexpectedly, knockdown of TIF1β promoted transcription even without PERM1 overexpression. These results suggest that the transcriptional regulators PGC-1α, BAG6, and KANK2 are required for PERM1-induced transcriptional activation *via* ERRE to some extent. To test if the recruitment of PERM1 to gene promoters has a regulatory role in transcription, we used mammalian one-hybrid system with *Gal4*-DNA binding domain fused with PERM1 and UAS-luc. Artificial recruitment of PERM1 to a gene promoter activated the transcription of UAS-luc, which was blunted by silencing of either PGC-1α, BAG6, or KANK2 (Figure 7C). Whether BAG6 or KANK2 regulates ERR target gene expression is unknown. We examined if silencing BAG6 or KANK2 alters ERR target gene expression. Primary cultured cardiomyocytes were treated with either scrambled siRNA (siControl), siRNA-*Bag6* (siBag6), or siRNA-*Kank2* (siKank2) and gene expression analysis was performed. As shown in Figure 7D, siRNA-mediated BAG6 and KANK2 significantly decreased the mRNA levels of ERR target genes (*Mcad*; *Sdha*; *Sdhd*; *Fh1*; *Cycs*; *Ndufv1*; *Idh3a*, all *p* < 0.05), suggesting that BAG6 and KANK2 are involved in transcriptional control of ERR target genes. Of note, we previously demonstrated that Perm1 directly regulates *Ndufv1* expression (14). Knockdown of PGC-1α, ANKRD1, BAG6, KANK2, and TIF1β by siRNA in cardiomyocytes was verified by western blotting (Figure 7E). Taken together with the results shown in Figure 6B, in which PERM1 interacts with ERRα in the nucleus, these results suggest that PERM1 acts as a transcription coactivator of ERRα that functionally interacts with PGC-1α, BAG6 and KANK2 (Figure 7E).

## Discussion

This study has revealed that PERM1 ablation in mice leads to a reduction of cardiac function and energy reserve in association with downregulation of OXPHOS proteins and broad changes in substrate metabolism. Furthermore, we show for the first time that in cardiomyocytes PERM1 binds to ERRα, a key transcription factor that regulates genes important for energy homeostasis. The recruitment of PERM1 to a gene promoter led to the activation of transcription (Figure 7C), indicating the role of PERM1 as a new transcription coactivator in the ERRα/PGC-1α axis in the heart. These findings position PERM1 as an important muscle-specific transcriptional regulator that coordinates cardiac function and energy metabolism, presumably *via* the ERRα/PGC-1α axis.

### Metabolic profile of PERM1-null hearts

*Perm1* deletion caused extensive alterations in substrate metabolism. Reduced energy reserve in association with downregulation of OXPHOS and upregulation of glycolysis pathway in *Perm*^–/–^ hearts recapitulates the metabolic phenotype of the failing heart (4, 8, 29). However, the reduced myocardial TG contents in *Perm1*^–/–^ mice contrasts the phenotype produced by pressure overload-induced heart failure in mice, where accumulation of TGs, DGs and MGs occurs, in part, due to the reduced fatty acid oxidation, in part, through downregulation of CPT1/2 (32). Of note, neither CTP1b nor CPT2 were downregulated in PERM1-null hearts (Supplementary Figure 2). The relative depletion of the global TG pool could explain decreased content of MGs and FFAs (Figure 4), leading to a relative fatty acid “starvation” in *Perm*^–/–^ mice. Given downregulation of CD36, which is localized to the plasma membrane and facilitates fatty acid uptake into muscle cells (Supplementary Figure 2B), it is plausible that cardiac fatty acid intake was persistently decreased in *Perm1* deficient mice.

In parallel to alterations in lipid metabolism, we observed accumulation of intermediates in the proximal part of glycolytic pathway (G6-P and F6-P, Figure 5). Despite upregulation of several glycolytic enzymes (OFK; BPGM; PGM, Figure 5), there is a significant downregulation of PDH, limiting the input to the TCA cycle. The glycolytic flux has to be diverted to bypass the bottleneck at the level of PDH. The diversion apparently occurs *via* the polyol pathway leading to remarkable increases in sorbitol and fructose content. Presumably, this flux is further directed to glycerol-3-phosphate (G-3P) synthesis (1.64-fold increase, *p* = 0.1, Figure 5) facilitated by upregulation on the cytoplasmic isoform of G-3P dehydrogenase (GPD1). The fact that the TG pool is decreased suggests, however, that the excess of G-3P is not utilized for *de novo* TG synthesis.

The most prominent change in metabolome of *Perm*^–/–^ hearts is the remarkable increase of sorbitol and fructose contents (Figure 4B). Increase in myocardial sorbitol and fructose is a signature of a diabetic heart, and it was recently shown that myocardial sorbitol and fructose content are directly correlated to the degree of diastolic dysfunction in diabetic patients (33). However, the causative link between the elevated levels of polyol pathway intermediates and contractile dysfunction is not well understood. Polyol pathway-mediated oxidative stress has been implicated in dysregulation of myocardial Ca^2+^ cycling, leading to contractile dysfunction (34). Fructose accumulation can also lead to increased formation of methylglyoxal, which reacts with arginine and lysine residues to form irreversible carbonyl adducts. Glycation of proteins by methylglyoxal was implicated in mitochondrial dysfunction and oxidative stress (35). Finally, channeling fructose excess into *de novo* TG synthesis may lead to enhanced cardiac lipid droplet formation (steatosis), which has been shown to be an independent predictor of diastolic dysfunction in type 2 diabetic patients (36). An interesting metabolic signature of *Perm*^–/–^ mice, which sets it apart from the diabetic heart, is a large accumulation of polyol pathway intermediates amid a global *decrease* in myocardial TG pool. It would be interesting to address whether *Perm*^–/–^ hearts are tolerant to high-fat diet through limiting lipid storage and oxidation. Future studies should address a possible role of PERM1 in pathological metabolic remodeling and related contractile dysfunction in the diabetic heart.

### Decrease in PCr/ATP ratio

The ratio of myocardial PCr to ATP is a sensitive index of the energetic state of the heart. PCr serves as a fast-exchange reserve of high-energy phosphates, the energy currency with the highest liquidity in the heart. Whenever energy demand exceeds energy supply, PCr levels decline first, and ATP decreases only when PCr is substantially depleted (37). PCr/ATP ratio is reduced during and after acute hypoxia (38) and in patients suffering from type 2 diabetes and various forms of heart failure (39). *Perm1*^–/–^ mice exhibit a significant decrease in PCr/ATP ratio as compared to WT mice (Figure 2), indicating a relative “energy starvation.” It should be noted that this observation may be confounded by the procedure of the heart harvesting necessary to perform high-energy phosphate profiling. With the best effort made, it took within 30 sec to transfer the tissue sample from the state of full oxygenation to the state of the full arrest of metabolic activity. This time may be sufficient to create a hypoxic challenge and contribute to PCr depletion. However, the conditions of tissue harvesting were exactly the same in *Perm1*^–/–^ and WT groups. Hence, whereas a potential hypoxic event cannot be excluded, the relative energy starvation in *Perm*^–/–^ mice would still be indicative of an energetic vulnerability specific to *Perm1* deletion. This raises a possibility that *Perm1*^–/–^ mice exhibit a reduced tolerance to acute hypoxia and/or increase in cardiac workload, which should be tested in future studies. The mechanism of the relative energy starvation in *Perm1*^–/–^ heart cannot be exactly defined at this point and may be a result of multiple factors. A computational study addressing possible factors contributing to a reduction in PCr/ATP ratio highlighted the 3 most important factors controlling this ratio: the activity of adenine nucleotide translocase, substrate dehydrogenation, and the activity of cytochrome oxidase (Complex IV). In this model, substrate dehydrogenation is a collective term of all processes supplying NADH to the electron-transport chain (ETC). In the heart, the main supplier of NADH to ETC is FAO. Of interest, cytochrome oxidase becomes the strongest controlling factor under conditions of hypoxia (40). Our proteomics analysis detected total of 17 proteins related to Complex IV. Out of those, four (COX6B1, COX7A1, COX8B, CMC1) were significantly downregulated, and others were unchanged. Whether such selective downregulation is sufficient to reduce the overall Complex IV activity, remains to be investigated. Nevertheless, the relative energy starvation in *Perm1*^–/–^ mice may be due to downregulation of FAO and/or downregulation of proteins involved in Complex IV.

The total ATP contents remain relatively stable and preserved in the stage of compensated hypertrophy (41), and it becomes significantly reduced only in advanced stages of heart failure (2, 42). Together with the moderate downregulation of OXPHOS proteins and upregulation of glycolysis (Figure 3), our data suggest that metabolic profile in the *Perm1* deficit heart mimics the metabolic remodeling at the early stage of heart failure. If this is true, maintaining PERM1 expression during pressure overload might prevent energy imbalance and cardiac dysfunction. This will be investigated in our future study.

### PERM1 acts as a transcription regulator in the ERRα/PGC-1α axis

The PGC-1/ERR transcriptional module has been established as a center of metabolism regulation in the heart. PERM1 was discovered by Kralli’s group as a muscle-specific protein that is induced by ERRs and PGC-1α/β in C2C12 myoblasts (15). It was initially reported that PERM1 regulates the expression of selective PGC-1/ERR target genes through unknown mechanisms (15). Consistent with conserved putative nuclear localization signals in the C-terminus of PERM1 (15), we found that PERM1 is localized to the nucleus in cultured cardiomyocytes (14). In this study, we show that PERM1 interacts with ERRα in the nucleus of the mouse heart (Figure 6), suggesting that PERM1 is a transcription partner of ERRα. Recent study by Cho et al. showed that PERM1 interacts with PGC-1α in the mouse heart and U2OS cells (cell lines from human bone osteosarcoma epithelial cells) (31). We confirmed the interaction of PERM1 with PGC-1α in cardiomyocytes (Figure 7). Transactivation of the ERRE by PERM1 through ERRα and PGC-1α (Figure 7) further suggests that PERM1 forms the functional transcription complex with ERRα and PGC-1α. Given that the predicted sequence of PERM1 contains ∅XXLL-type motif in the N-terminus that is often involved in protein-protein interaction but there is no other obvious protein motifs suggestive of a molecular function (15), it is plausible that PERM1 plays a role as an adaptor protein that promotes recruitment of transcription regulators to the ERRE to initiate transcription. It is interesting to note that the promoters of both *Perm1* and *ERR*s contain the ERRE (15, 43). We speculate that the PERM1/ERRα/PGC-1α transcriptional complex acts as a feedforward mechanism to dramatically boost transcription of ERRα and its target genes involved in OXPHOS in response to increased energy demands in the heart. This needs to be investigated in our future study.

MS-based unbiased screening of PERM1-bound proteins in cardiomyocytes identified several candidates that may be involved in the transcription regulation by PERM1 (Figure 7). Among them, we found that BAG6 (also known as BAT3) and KANK2 (also known as SIP) are required for PEMR1 to transactivate the ERRE (Figure 7). BAG6 is a nuclear protein, and recent study suggests that BAG6 may act as a component of some chromatin regulator complex that regulates histone 3 lysine 4 dimethylation (H3K4me2) (44), which generally leads to gene activation. Given that PERM1 requires BAG6 to activate the ERRE (Figure 7), it is plausible that PERM1 also recruits epigenetic modulator(s), presumably for chromatin remodeling of ERR/PGC-1 target genes. A cardioprotective effect of KANK2 against myocardial infarction through decreasing collagen deposition and apoptosis has been reported (45). KANK2 is also known to be involved in transcription regulation and interacts with steroid receptor co-activator 1 (SRC-1) in the human heart (46), which facilitates the transcription initiation mediated by nuclear receptors. However, the role of KANK2 in transcriptional control through the ERR/PGC-1 axis needs to be further investigated.

The current study was aimed at elucidating the role of PERM1 as a transcriptional regulator. Bock et al. recently demonstrated that PERM1 is localized in mitochondrial outer membrane that interacts with the MICOS (mitochondrial contact site and cristae organizing system)-MIB (mitochondrial intermembrane space bridging) complex and connects the mitochondria and sarcolemma (47). Our current study and the other studies also confirmed the localization of PERM1 in the mitochondria of cardiac tissue as well as in the nucleus and cytosol (31) (Supplementary Figure 3). Consistently, our LC-MS/MS-based screening of PERM1-bound proteins in cardiomyocytes (whole lysates) detected the components of MICOS-MIB complex, such as MIC60 and SAMM50 (Supplementary Dataset 8). Overall, our current study and the other studies (31, 47) suggest that PERM1 can regulate mitochondrial function through both transcription dependent and independent mechanisms.

### ERRα/PGC-1α transcriptional regulation in heart failure

This study demonstrates that Perm1 functionally interacts with PGC1α and ERRα. Impaired ERRα/PGC-1α pathways are associated with metabolic dysfunction in the failing heart. Downregulated genes important for energy metabolism in the failing heart are mostly recognized as the ERR/PGC-1 target genes (4, 8, 48). Genetic deletion of either PGC-1α or ERRα leads to global downregulation of OXPHOS genes in the heart (4, 7, 18, 43). However, neither ERRα-KO nor PGC-1α-KO mice (systemic, constitutive) exhibit any change in cardiac function (4, 7). Of note, cardiac-specific PGC-1α-KO mice develop cardiac dysfunction at the age of 17 weeks (-29% in EF), in association of downregulation of FAO proteins (CPT1b, FATP) as well as glycolysis enzymes (GLUT4; PFKm) (49). Downregulation of ERRα occurs early in response to pressure overload in mice (7 days of TAC), whereas there is no change in the expression of ERRγ (4). As discussed in our review (12), a variability of outcomes in PGC-1α expression has been reported in the human and mouse failing hearts: downregulation (8, 50–54); unchanged expression (4, 18, 55, 56); and upregulation (10). In addition, impaired mitochondrial biogenesis was observed in the human and mouse heart failure even when PGC-1α expression was unchanged (29). Thus, the changes in the expression and the activity of PGC-1α alone cannot completely explain metabolic reprogramming in the diseased heart. More importantly, maintaining PGC-1α expression during pressure overload in mice failed to rescue contractile function in this setting. (56, 57) To date, it remains unknown whether maintenance or overexpression of ERRα has any beneficial effect for cardiac function under conditions of hemodynamic stress. Our study demonstrated that both mRNA and proteins levels of Perm1 were significantly reduced in the human and mouse failing heart (14). More importantly, the initial response to phenylephrine-induced hypertrophy in cardiomyocytes, which mimics metabolic changes in pressure overload-induced heart failure, occurred in the reduced mRNA level of Perm1, followed by downregulation of ERRα an PGC-1α (14). Thus, it is possible that downregulation of Perm1 is an early precursor of metabolic remodeling under pathological stress. If so, maintaining Perm1 expression might attenuate the impairment of the ERRα/PGC-1α axis during pressure overload and prevent metabolic perturbations and the progression of heart failure at the early stage. This needs to be determined in our future study.

In conclusion, this study establishes the role of PERM1 as a new transcriptional regulator in the heart that maintains energy reserve and contractile function through interacting with ERRα and PGC-1α. The therapeutic potential of PERM1 in heart failure and cardiac metabolic syndromes should be tested in future studies.

## Data availability statement

The original contributions presented in this study are included in the article/Supplementary material, further inquiries can be directed to the corresponding author.

## Ethics statement

This animal study was reviewed and approved by University of Utah and the Rutgers New Jersey Medical School Institutional Animal Care and Use Committee.

## Author contributions

JW and SO contributed to the initial design of study and manuscript preparation. TS performed echocardiography. SA, SY, AJ, KGS, KO, AS, and AH performed gene expression study. KS, KC, and SAO performed western blotting analysis. MT performed EM analysis. SO, JB, XX, SK, and YM prepared primary cultured neonatal cardiomyocytes and performed western blotting analysis, reporter gene assays, and DNA binding assays. DC and TOL contributed to the echocardiography analysis. TOL and HL performed LC/MS-MS analyses. JC performed GCMS analysis. JM performed lipidomic analysis. YS contributed to the mouse study. SD supervised echocardiography analysis and edited the manuscript. All authors contributed to the article and approved the submitted version.
