# Supplementary material for: PERM1 regulates energy metabolism in the heart via ERRα/PGC−1α axis
Source: Front Cardiovasc Med. 2022 Nov 7;9:1033457. doi: 10.3389/fcvm.2022.1033457 (PMC9676655; doi:10.3389/fcvm.2022.1033457)
Supplement: Supplementary file 11 [file Table_11.docx]

**Online Supplementary Material**

**PERM1 regulates energy metabolism in the heart via ERRα/PGC-1α axis**

Shin-ichi Oka^1^, Karthi Sreedevi^2^, Thirupura S. Shankar^3^, Shreya Yedla^2^, Sumaita Arowa^2^, Amina James^2^, Kathryn G. Stone^2^, Katia Olmos^2^, Amira D. Sabry^3^, Amanda Horiuchi^3^, Keiko M^3^. Cawley, Sean A. O’very^3^, Mingming Tong^1^, Jaemin Byun^1^, Xiaoyong Xu^1^, Sanchita Kashyap^1^, Youssef Mourad^1^, Omair Vehra^1^, Dallen Calder^3^, Ty Lunde^3^, Tong Liu^4^, Hong Li^4^, J. Alan Mashchek^5^, James Cox^5,6^, Yukio Saijoh^3^, Stavros G. Drakos^3,7^, Junco S. Warren^2,8,9^

^1^Department of Cell Biology and Molecular Medicine, Rutgers New Jersey Medical School, Newark, NJ, USA

^2^Fralin Biomedical Research Institute at Virginia Tech Carilion, Virginia Tech, Roanoke, VA, USA

^3^Nora Eccles Harrison Cardiovascular Research and Training Institute, University of Utah, Salt Lake City, UT, USA

^4^Center for Advanced Proteomics Research, Department of Microbiology, Biochemistry, and Molecular Genetics, Rutgers New Jersey Medical School and Cancer Institute of New Jersey, Newark, NJ, USA.

^5^Metabolomics Core Research Facility, University of Utah, Salt Lake City, United States

^6^Department of Biochemistry, University of Utah, Salt Lake City, United States

^7^Division of Cardiovascular Medicine, University of Utah School of Medicine, Salt Lake City, UT, USA

^8^Center for Vascular and Heart Research, Fralin Biomedical Research Institute, Virginia Tech, Roanoke, VA, USA

^9^Department of Human Nutrition, Food and Exercise, Virginia Tech, Blacksburg, VA, USA

Running Title: Role of PERM1 in the heart

Address for correspondence:

Junco S. Warren, PhD.

Fralin Biomedical Research Institute

Virginia Tech Carilion

4 Riverside Circle,

Roanoke, VA, 24012

Phone: (540) 526-2293

Email: juncowarren@vtc.vt.edu

Keywords: PERM1, heart, energy metabolism, ERRα, ERRE, transcription

Category: heart, energy metabolism, heart failure, myocytes, oxidative phosphorylation, proteomics, metabolomics

Issue Section: Original Article

**METHODS**

**Animals and Tissue Harvest**

*Perm1*-KO mice were generated by Crispr/Cas9 system with gRNA targeting at the exon 2 in the Sadoshima lab at the Rutgers New Jersey Medical School. The *Perm1*-KO allele lacks 1157 bp in exon 2 and induces frame shift and an artificial stop codon. Genotyping of WT and mutant mice was performed using the primers, which target the Crispr target region. Primer sequence is described below. In terminal study, mice were anesthetized with the lethal dose of pentobarbital (200 mg/kg) via intraperitoneal injection, and the heart was the isolated during the deep anesthesia. Tissue samples from ventricles were immediately frozen in liquid nitrogen and stored at -80°C until they were used for analysis.

**Primer sequence for genotyping**

The primers to detect mutate allele are:

LEFT PRIMER: tgatgtgcaatggacaacttcc

RIGHT PRIMER: CTTTCTTCTTCTTGGGGGCTTG

The primers to detect WT allele are:

LEFT PRIMER: CAGGCTCAGAGATCAAGCCAGA

RIGHT PRIMER: CTTTCTTCTTCTTGGGGGCTTG

**Transverse Aortic Constriction Surgery**

Constriction of the transverse thoracic aorta (TAC) was performed on 3-month-old WT male mice at the Rutgers New Jersey Medical School, as previously described^1,2^. Thirty minutes before surgery a single dose of ZooPharm Buprenorphine SR (sustained release) (0.15mg/Kg; 72 hour efficacy) was administered subcutaneously. Mice were anesthetized with isoflurane (2% in O2) through nosecone, which was delivered from a VetFlo Vaporizer. During anesthesia and surgery, sterile eye ointment was used to protect the cornea from becoming dry during surgery. When a surgical plane of anesthesia was achieved (~5 minutes) and confirmed with lack of pedal reflex, a topical depilatory agent was applied to the neck. Once the hair has been sufficiently removed with the topical depilatory agent, any excess was removed using sterile water. The depilatory did not remain on the skin for more than one minute to avoid irritation. The underlying skin was then disinfected with alternating applications of betadine and 70% alcohol. During the first two applications betadine scrub was used and on the third application betadine solution was applied and allowed to dry before any incisions are made. Lidocaine (7mg/kg) was then introduced under the skin at the incision site. Mice were placed supine and temperature was maintained at 37C with a water circulating heating pad. A incision ~0.5cm in length was made at the level of the suprasternal notch (Incision orientation is from head to toe). Blunt dissection under a low powered microscope allowed for visualization of the aortic arch. A ligation clip applicator was calibrated to the appropriate gauge needle for the weight of the mouse and a 1/1 titanium microclip (Horizon) is placed at the aortic arch between the innominate artery and the left common carotid artery. The advantage of this novel surgical approach versus conventional approaches for TAC was that the chest cavity was not breached, the animal did not develop a pneumothorax and does not require intubation and ventilation. The absence of a chest wall incision also ensured faster recovery and less pain. The muscle, sternum and skin were closed with 5-0 dermalon sutures. Mice were allowed to recover on a warming pad until they were fully awake. The mouse was returned to its cage when fully awake. The sham procedure is identical except that the aorta was not ligated. Following surgery mice were monitored every 6-12 hours in the animal facility. After 72 hours if the animal was showing signs of distress, buprenorphine (regular release) was administered at 8-hour intervals for up to 48 hours as needed. The determination for subsequent doses was made in conjunction/consultation with veterinary staff. In addition to buprenorphine SR, the NSAID carprofen was given subcutaneously after induction of anesthesia and redosed once every 24 hours for 72 hours post-op.

**Histology**

Whole hearts were rapidly excised from WT and *Perm1^-/-^* mice at the age of 10 weeks and were fixed in 4% paraformaldehyde and then embedded in paraffin. Hearts were then sectioned at 4 μm and placed on slides. Hematoxylin and eosin (H and E) (Sigma) staining was performed according to the manufacturer’s protocols^3^. Tissue sections were visualized with imaged on Nikon eclipse TE2000-U microscope with SPOT software (Diagnostic Instruments).

**Metabolomics**

Ventricular samples from WT and Perm1^-/-^ hearts were analyzed using two metabolomic platforms [GC-TOF MS and hydrophilic interaction chromatography (HILIC)-quad-time of flight (QTOF)/MS] for untargeted metabolomic screening, as previously performed^3^. Briefly, the freeze-clamped cardiac tissue samples (~15 mg) were homogenized in the extraction solution (methanol/water (8:1) with d4 succinate and d27 myristic acid) using a beat ruptor (Omini) and ceramic bead (speed: 6.15, time: 30 sec). Those homogenates were incubated for one hour at -20°C. After centrifugation (13,000 x g at 4°C for 10 min), the supernatant was centrifuged to remove large molecules. Samples were maintained at 4ºC throughout the extraction process. The extraction samples were dried using speed-vac (MiVac Duo, Barnstead/Genevac Inc., Gardiner, NY) overnight. All GC/MS analysis was performed with a Waters GCT Premier mass spectrometer fitted with an Agilent 6890 gas chromatograph as previously performed^3,4^. Briefly, a Gerstel MPS2 autosampler was employed in conjunction with a CIS4 cold injection inlet. Dried samples were suspended in 40 μL of a 40 mg/mL O-methoxylamine hydrochloride in pyridine and incubated for one hour at 30°C followed by the transfer of 25 uL to autosampler vials. Ten μL of MSTFA was added by the autosampler and incubated for 30 minutes at 37°C with shaking. One μL of sample was injected to the cool inlet with the following program, 95°C for 30 seconds followed by a 10°C/second ramp to 250°C and a hold time of 3 minutes. The gas chromatograph held at an initial temperature of 95°C for one minute followed by a 40°C/min ramp to 110°C and a hold time of 2 minutes. This was followed by a second 5°C/min ramp to 250°C then a third ramp to 350°C with a final hold time of 3 minutes. A 30 m Restek Rxi-5 MS column with a 5 m long guard column was employed for analysis. *Data analysis:* Data were collected by MassLynx 4.1. Initial analysis of known metabolites was performed using QuanLynx with data transfer to Excel. Peak picking was performed using MarkerLynx with data mining performed using SIMCA-P ver. 12.0.1. The values were normalized by wet tissue weight.

**Bioinformatics.** The PCA and fold-change plots of the metabolomic data were carried out using MetaboAnalyst 5.0 software^5^. Differences in the abundance of individual metabolites were determined using a Student’s *t* test (two-tailed, unpaired comparison). A value of *P* < 0.05 was considered statistically significant. Data are given as mean ± SEM.

**Lipidomics**

Agilent 6490 triple quadrupole (QqQ) mass spectrometry was used to perform lipidomic profiling of WT and Perm1^-/-^ hearts, as described in our previous publication^6^.

**Chemicals.** LC-MS-grade solvents and mobile phase modifiers were obtained from Honeywell Burdick & Jackson, Morristown, NJ (acetonitrile, isopropanol, formic acid), Fisher Scientific, Waltham, MA (methyl *tert*-butyl ether) and Sigma–Aldrich/Fluka, St. Louis, MO (ammonium formate, ammonium bicarbonate). Lipid standards Mouse SPLASH LIPIDOMIX and Deuterated Ceramide LIPIDOMIX were obtained from Avanti Polar Lipids (Alabaster, AL) and labeled carnitine standards (NSK-B and NSK-B-G) were obtained from Cambridge Isotope Laboratories (Cambridge, MA).

**Sample Preparation.** Extraction of lipids was carried out using a biphasic solvent system of cold methanol, methyl *tert*-butyl ether (MTBE), and water ^6^with some modifications. In a randomized sequence, tissue lipids were extracted in bead-mill tubes (ceramic 1.4 mm, Mo-Bio, Qiagen, Germantown, MD) containing a solution of 225 µL MeOH, 750 µL methyl tert-butyl ether (MTBE), internal standards (Avanti Mouse SPLASH LIPIDOMIX, Deuterated Ceramide LIPIDOMIX, and labeled carnitine standards (NSK-B and NSK-B-G) all at 10 µL per sample. Samples were homogenized in one 30 sec cycle then rested on ice for 1 hr with occasional vortexing. Then 188 µL of PBS was added followed by a brief vortex. Samples were then centrifuged at 14,000 x *g* for 10 minutes at 4 °C, and the upper phases were collected. Another aliquot of 750 µL MTBE was added to the bottom aqueous layer followed by a brief vortex. Samples were then centrifuged at 14,000 x *g* for 10 minutes at 4 °C, the upper phases were combined and evaporated to dryness under speedvac. Lipid extracts were reconstituted in 250 µL of mobile phase B and transferred to an LC-MS vial for analysis. Concurrently, a process blank sample was prepared and then a pooled quality control (QC) sample was prepared by taking equal volumes (~50 µL) from each sample after final resuspension.

**LC-MS Analysis.** Lipid extracts were separated on an Acquity UPLC CSH C18 1.7 µm 2.1 x 100 mm column maintained at 65°C connected to an Agilent HiP 1290 Sampler, Agilent 1290 Infinity pump, and Agilent 6490 triple quadrupole (QqQ) mass spectrometer. Sphingolipids were detected using dynamic multiple reaction monitoring (dMRM) in positive ion mode. Source gas temperature was set to 150°C, with a gas (N_2_) flow of 17 L/min and a nebulizer pressure of 20 psi. Sheath gas temperature was 200°C, sheath gas (N_2_) flow of 10 L/min, capillary voltage is 3500 V, nozzle voltage 500 V, high pressure RF 190 V and low pressure RF was 120 V. Injection volume was 10 µL and the samples were analyzed in a randomized order with the pooled QC sample injection eight times throughout the sample queue. Mobile phase A consists of ACN:H_2_O (60:40 *v/v*) in 10 mM ammonium formate and 0.1% formic acid, and mobile phase B consisted of IPA:ACN:H_2_O (90:9:1 *v/v/v*) in 10 mM ammonium formate and 0.1% formic acid. The chromatography gradient started at 15% mobile phase B, increased to 30% B over 0.7 min, increased to 60% B from 0.7-1.4 min, increased to 80% B from 1.4-7.0 min, and increased to 99% B from 7.0-7.14 min where it was held until 10.2 min then returned to starting conditions at 10.3 min. Post-time was 4 min and the flowrate was 0.4 mL/min throughout. Collision energies and cell accelerator voltages were optimized using sphingolipid standards with dMRM transitions as [M+H]^+^→[*m/z* = 284.3] for dihydroceramides, [M-H_2_O+H]^+^→[*m/z* = 264.2] for ceramides, [M-H_2_O+H]^+^→[*m/z* = 271.3] for isotope labeled ceramides. All carnitines were monitored with dMRM transitions as [M+H]^+^→[*m/z* = 85.1]. Sphingomyelins and phosphatidylcholines were monitored with dMRM transitions as [M+H]^+^→[*m/z* = 184.1]. Glycerolipids were monitored with dMRM transitions as [M+NH_4_]^+^→[m/z = neutral loss of acyl chain]. Lipids without available standards were identified based on HR-LC/MS, quasi-molecular ion and characteristic product ions. Their retention times were either taken from HR-LC/MS data or inferred from the available sphingolipid standards. LC-MS Data Processing. For data processing, Agilent MassHunter (MH) Workstation and software packages MH Qualitiative and MH Quantitative were used. The pooled QC (n=8) and process blank (n=4) were injected throughout the sample queue to ensure the reliability of acquired lipidomics data. Data exported from MH Quantitative was evaluated using Excel where initial lipid targets were parsed based on the following criteria. Only lipids with relative standard deviations (RSD) less than 30% in QC samples were used for data analysis. Additionally, only lipids with background AUC counts in process blanks that were less than 30% of QC were used for data analysis. The parsed excel data tables were normalized based on the ratio to class-specific internal standards, then to tissue mass prior to statistical analysis. Statistical Analysis and Data Visualization. Multivariate analysis was performed using MetaboAnalyst^5^. Statistical models were created for the normalized data after logarithmic transformation (base 10) and Pareto scaling. Initial pass for the volcano plot used a fold change (FC) cut off of 1.5 and raw p-value cut off: 0.05.

**Proteomic analysis of whole lysates**

Proteomic analysis of ventricular tissue collected from *Perm1^-/-^* mice and WT littermates at the age of 10 weeks were conducted using label-free quantitative LC-MS/MS, as described in our previous publication^7^, with some modifications. PERM1-bound proteins were also analyzed in the LC-MS/MS system. Label-free quantitation of protein expression was accomplished using the PEAKS 7.5 Studio software. The significance of changes in proteins was determined by a Student’s t-Test of WT and KO areas. Proteins identified by at least 1 unique peptide and 2 or more total peptides, with a p-value less than 0.05, were considered significant. Enrichment analyses of the proteins that were significantly changed by loss of Perm1 were performed using the STRING database, as previously demonstrated^8^.

**Sample preparation.** Protein extraction from heart tissue was carried out by lysing the tissue in lysis buffer (0.1 M Tris/HCL pH 7.6, 4% SDS, 0.1M DTT) and then sonicating the samples with a tip sonicator for 3 cycles of 10 seconds and incubating at 95 °C for 10 minutes. The lysate was then centrifuged at 16,000 g for 15 minutes to remove any insoluble material. Lysate (200 μL) from each sample was loaded into a Vivacon 500 filter units, concentrated (10-fold) at 13000 g and then washed three times with 100 μL of UA buffer (8M Urea, 0.1M Tris/HCl pH 8.5). The concentrate was mixed with 100 μL of 50 mM iodoacetamide in UA buffer and incubated in darkness at room temperature for 20 min followed by centrifugation for 15 min (13,000g). The concentrate was then washed twice with 100 μL of UA buffer followed by two washes with 100 μL of 50 mM ammonium bicarbonate. Approximately 400 μg of protein from each sample was mixed with trypsin (1:80) in the filter and incubated overnight at 37 °C. The peptides were then eluted with 50 mM ammonium bicarbonate and acidified with 1% formic acid.

**LC-MS/MS Analysis.** Samples were diluted in 50 µL of 0.1 % formic acid, and the peptides were separated on a 15 cm long, 75 μm ID reversed-phase PicoFrit column packed with 3 µm Reprosil-Pur C-18 resin, using a 210-min (from 5-30% acylcarnitine in 180 min and 30-45% acylcarnitine in 30 min) gradient. The MS/MS analysis was carried out on an LTQ-Orbitrap Velos Pro instrument (Thermo Fisher Scientific). The MS1 scans were acquired in the Orbitrap analyzer with 30,000 resolution at m/z of 400. The AGC targets were set at 1 × 10^6^ for MS1 scans 3 × 10^4^ for MS2 scans. The 20 most intense ions in each full MS1 scan were fragmented using CID. Dynamic exclusion was enabled with repeat count of 1 and exclusion duration of 60 sec.

**Data analysis.** Raw MS files were processed with MaxQuant version ^9^ 1.4.1.2, for identification against the Uniprot Canine database. The search was carried out using the Andromeda search engine built in MaxQuant. The search included variable modifications for oxidation of methionine, acetylation of protein n-termini and fixed modification of carbamidomethylation of cysteine. The mass tolerance for MS1 and MS2 searches was 20 ppm and 0.5 Da, respectively. A false discovery rate of 1% was used for both proteins and peptides and only peptides with more than 6 amino acid residues were considered for identification and quantitation. Label free quantitation was carried out in MaxQuant using 3 technical replicates for each biological sample. During label free quantitation the isotopic patterns for each feature was matched across multiple runs using peptide identification, high mass accuracy and nonlinear remapping of retention times. Data was normalized using compiled peptide intensities and pair wise peptide ratios were calculated across the runs for all unique peptides mapping to a protein and displayed as an LFQ intensity value ^10^. The complete data set for myocardial proteomics is presented in Dataset S3.

**Mass-spectrometry analysis of PERM1-bound proteins**

*Perm1*-Flag was expressed in primary cultured cardiomyocytes with adenovirus vector (10cm dish). After 2 days of transduction, cells were lysed with 500 µl lysis buffer (50 mM Tris, pH7.6, 1% Triton-X100, 10 mM EDTA, 150 mM NaCl, 50 mM NaF, 10 mM Sodium Butylate, 0.1 mM dithiothreitol). *Perm1*-Flag was immunoprecipitated with anti-Flag antibody (Sigma, A2220). The immune-complex was subjected to SDS-PAGE followed by mass spectrometric analysis, Briefly, the peptides that resulted from digested proteins by trypsin were desalted using a C18 Spin Cartridge (Pierce) and further separated on an Ultimate 3000 Nano UHPLC System (Thermo Fisher Scientific) using a C18 reversed-phase nano column (Acclaim PepMap RSLC, 75 μm × 50 cm, 2 μm, 100 Å, Thermo Fisher Scientific) with a 100 min binary gradient. The eluted peptides were then directly introduced an Orbitrap Fusion Lumos Tribrid MS system with a spray voltage of 2kV and a capillary temperature of 275°C. The MS scan range is m/z of 375-1500.  The automatic gain control (AGC) target were set to 5 × 10^5^ for MS scan in Orbitrap and 1 × 10^4^ for MS/MS scan in Ion Trap mass analyzer. The precursor was selected within a 1.2 m/z isolation window and fragmented using Higher Energy Collision Dissociation (HCD) fragmentation at Collision Energy set at 30%. The MS/MS spectra were searched against the UniProt Rat database using Sequest search engine on Proteome Discoverer (V2.4) platform. The MS error tolerance set to 10 ppm and an MS/MS error tolerance of 0.5 Da. The protein false discovery rate is less than 1%. The protein relative quantitation was compared based on label free quantitation (LFQ) method.

**High-energy phosphate profiling**

Standard stock solutions were freshly prepared on the day of experiments (ATP, ADP, AMP, PCr, creatine, and NAD, all from Sigma, St Louis, MO, USA), and dilution was made by adding mobile phase immediately before each assay, which was used as references for peaks quantification. The cocktail of these standards was run before and after actual samples to make sure that the system performance did not decline. Approximately 10 mg of frozen wet tissue was homogenized in 120 μl of 0.4 M perchloric acid using a ceramic bead tube kit (MO BIO Laboratories, Inc., Carlsbad, CA) and Bead Ruptor (Omini International, Kennesaw, GA), which provides ultra-rapid shaking. After precipitation with 1M KOH the extracts were centrifuged for 3 min at 4°C (13,000 g). The supernatant was centrifuged at 4 °C twice (14,000 g). Twenty microliters of the extracted sample were manually injected to the Shimadzu HPLC system with SPD-20 and LC-20 Pumps.

**Transmission electron microscopy (TEM)**

TEM and mitochondrial analysis were performed as described previously^11^. In brief, WT and Perm1-KO mice were sacrificed at 4 months. The hearts were isolated for EM analysis. The tissue samples were fixed in EM buffer (Electron Microscopic Sciences). For morphometric analysis of mitochondrial cristae, cristae were measured using the image J Multimeasure plug-in. Briefly, cristae connected or disconnected with mitochondrial membrane were calculated, and number of cristae in each mitochondrial were counted to calculate cristae density in each mitochondria.

**Chromatin immunoprecipitation (ChIP) assay.**

Heart tissue, cultured cardiomyocytes, were cross-linked with formaldehyde. The nuclear fraction was isolated and sonicated to generate a chromatin solution that was then used for immunoprecipitation. Primers used for investigating mouse genome are listed in online Supplement as follows: (5’ to 3’). Cpt2 - CCCTGTGGGCGGAGTTGAACT and ATTTTGTCCGTGACCTTCGCGC; Mcad *-* ATCTAGCCCAGAATTTGTTGTTCCAGTG and GCGGTGGCTGAGGGAGTTCC; Idh3a - TGATGGATGGCGGAGGCGAGC and TGGGGACACCCGGAGCAGTAC; Sdhd - CCTTCCGGTTCACGCTTCAGGT and TCGCTTTCGAGGGCTCAAGGT; ERRα - CTCGTGTCTCACCTCTGCCTTT and GACACTTGACAGATTTGGTAGATCAG; Ndufv1 - GCTCCCCACCCCGTGTGTTT and GGGAGCCAGACGGGTCACCT; and Ndufs1 - CTTATATAGAGACGGAGGCGTTTC and GACGACCTGTGGGCCCTTTAGTTA. The corrected chromatin fragment was validated by qPCR with Maxima SYBR Green qPCR master mix (Fermentas).

***In vitro* DNA binding assay.**

Biotin-labeled *Idh3a* promoter (380 bp) was prepared by PCR with a biotin-labeled primer. Endogenous ERRα was knocked down in primary cultured cardiomyocytes by siRNA transfection. After 2-3 days of transfection, the cells were lysed with 300 μl of a nuclear lysis buffer [25 mM Hepes, pH7.9, 0.1 mM EDTA, 10 mM Sodium Butyrate, 10 mM NaF, 1 mM DTT, 1 % NP-40, 420 mM KCl, 1xProteinase inhibitor cocktail]. The 100 μl lysate was diluted with 300 μl of a dilution buffer [10 mM Hepes, pH 7.9, 2.5 mM MgCl2, 5 % Glycerol, 1 mM DTT, 0.1%NP-40]. The total 400 μl of diluted lysate was incubated with 1 μg biotin-labeled DNA and streptavidin-beads at 4 °C for 2-4 hours with rotation. The protein-DNA complex was washed with 1 ml of a washing buffer [10 mM Hepes, pH 7.9, 2.5 mM MgCl 2, 5 % Glycerol, and 50 mM KCl 1 mM DTT, 0.1%NP-40] for 3 times. The protein bound to the DNA was analyzed with Western blot analyses.

**Luciferase reporter gene assay.**

Luciferase assays were performed in primary cultured rat cardiac myocytes as previously performed (see the details in online supplement). Reporter plasmids (0.3 μg per well), including 3xERRE-luc and UAS-luc, mammalian expression vectors (0.7 μg per well), including pDC316 control vector, pDC316-Perm1 and pFA-Perm1, and siRNA (20 pM) against PGC-1α, ERRα, Ankrd1, Bag6, Kank2 and TIF1β, were transfected into cells plated in 12 well plates using LipofectAmine 2000 (Invitrogen). Total plasmids were kept at 1 μg per well using pDC316 control vector. The total siRNA was kept 20 pM per well using control siRNA. Two μl LipofectAmine 2000 was used per well. Fifty ul OPTI-MEM was used for dilution of plasmid vectors and of LipofectAmine 2000, respectively. Luciferase assays were performed 2 days after transduction with a luciferase assay system (Promega).

**Primary cultures of neonatal rat ventricular myocytes.**

Primary cultures of ventricular cardiac myocytes were prepared form 1-day-Crl :(WI) BR-Wistar rats (Harlan) as previously described in our publication (REF). The details of methods are described in online supplement. A cardiac myocyte- and fibroblast-rich fraction was obtained by centrifugation through a discontinuous Percoll gradient. Cells were cultured in complete medium (CM) containing Dulbecco’s modified Eagles’s medium/F12 supplemented with 5 % horse serum, 4 μg/ml transferrin, 0.7 ng/ml sodium selenite, 2 g/l bovine serum albumin (fraction V), 3 mM pyruvate, 15 mM Hepes pH 7.1, 100 μM ascorbate, 100 mg/l ampicillin, 5 mg/l linoleic acid, and 100 μM 5-bromo-2’-deoxyuridine (Sigma). Culture dish were coated with 0.3 % gelatin.

***siRNAs.*** Small interfering RNA (siRNA) used for this study were obtain from Qiagen, including control siRNA (1022076), rat PGC-1α (S100271012), rat ERRα (SI01511055), rat Ankrd1 (SI01487731), rat Bag6 (SI00276955), rat Kank2 (SI05569522) and rat TIF1β (SI02048067). The siRNA was transfected with Lipofectamine RNAiMax (Invitrogen). Four and half μl of Lipofectamine RNAiMax was diluted with 125 μl OPTI-MEM. Two μl of 20 μM siRNA was diluted with 125 μl OPTI-MEM. The diluted Lipofectamine RNAiMax and siRNA was then mixed and incubated at room temperature for 20 minutes. The mixture was added to cardiomyocytes plated on a 1/6 well plate with 2 ml DMEM/F21 medium. The cells were used after 24-48 hours of transfection.

**Western blot analysis**

Mouse heart tissue was dissected and the left ventricle was lysed in a lysis buffer containing 50 mM Tris-HCl (pH 7.4), 150 mM NaCl, 50 mM NaF, 10 mM EDTA, 1 % Triton-X 100, 0.1 mM DTT and 1x Protease Inhibitor Cocktail (Sigma). Total protein lysates (10–30 µg) were incubated with SDS sample buffer [Final concentration: 100 mM Tris pH 6.8, 2 % SDS, 5 % glycerol, 2.5 % 2-mercaptoethanol, and 0.05 % bromophenol blue] at 95 °C for 5-20 minutes.

These denatured protein samples were transferred to polyvinylidene difluoride membranes and probed with antibodies against Perm1 (Sigma HPA032711), ERRα (Millipore ERR46Y), and PGC-1α (Millipore Ab3242).

**Gene Expression Analysis.**

Real-time PCR was performed using Taqman primers as described previously^3^. Briefly, RNA was extracted using TRizol (Life Technologies), followed by ethanol extraction. Reverse transcription was performed from 1ug of RNA using QuantiTect Reverse Transcription Kit according to the manufacture’s instruction (Qiagen). Taqman primers that were used in this study are listed below. The targeted genes were normalized by the gene expression level of *Tbp (*Mm00446971m1).

| Gene name | Protein name | Cat# |
| --- | --- | --- |
| *NPPA* | ANP | Mm01255747_g1 |
| *Myh6* | MHC-α | Mm00440359_m1 |
| *Myh7* | MHC-β | Mm00600555_m1 |
| *SGLT1/Slc2a1* | GLUT1 | Mm00441480_m1 |
| *SGLT4/Slc2a4* | GLUT4 | Mm00436615_m1 |
| *Cd36* | CD36 | Mm00432403_m1 |
| *Cpt1b* | CPT1b | Mm00487191_g1 |
| *Cpt2* | CPT2 | Mm00487205_m1 |
| *Acadm* | ACADM | Rn00566390_m1 |
| *Sdhd* | SDHD | Mm00546511_m1 |
| *Idh3a* | IDH3a | Mm00499674_m1 |
| *Ndufv1* | NDUFV1 | Mm00504941_m1 |
| *Ndufs8* | NDUFS8 | Mm00523063_m1 |
| *Cycs* | CYCS | Mm01621048_s1 |

As for knockdown study (Figure 7D), Real-time RT-PCR was performed using the Maxima SYBR Green qPCR master mix (VWR, 101414). β-actin was used as an internal control. The mean value from wild type mice was expressed as 1. PCRs were carried out using the following oligonucleotide primers (5’ to 3’): Mcad, GCAGATTTTCGGAGGCTATGGATTC and TTAATTTTTATACTTTTCAATGTGCTCACGAG; Sdha, CGCACTGCAGACCATATATGGTG and GGGTGTGCTTCCTCCAGTGTTC; Sdhd, CTGTGGTGGACTACTCTCTGGCT and CATCGTGGTAATTGAAGTAGCAAAGCCC; Fh1, GTTGGTCACAGCTCTTAATCCACATATAG and GAGATGCTTCAGTGTCCTTCACAGA; Cycs, ATTCTCTTACACAGATGCCAACAAGAACAA and AGCTATTAGGTCTGCCCTTTCTCC, Ndufv1, CTGTGGGAGATCAGCAAGCAGAT and GTGCTGCTGGGCAAATCGTTG; Idh3a, GGAGACATCCTTAGTGATCTGTGTG and ATGTCCTTGCCTGCAATGTCCG, and β-actin, AAGACCTCTATGCCAACACAGTGC and CACTTGCGGTGCACGATGGAG.

**Supplement Data**

**Supplement Table S1.** Echocardiography of WT, Perm1^+/-^, and Perm1^-/-^ mice

**Cellular Component**

**B**

Intracellular membrane-bounded complex

Mitochondrion

Mitochondrial envelop

Mitochondrial membrane

Mitochondrial protein complex

Organelle envelop

Cytoplasm

Inner mitochondrial membrane protein complex

**141**

**74**

**70**

**61**

**62**

**50**

**77**

**179**

**171**

**34**

Organelle inner membrane

Mitochondrial inner membrane

**47**

**Cellular Component**

**273**

**282**

**143**

**39**

**41**

**303**

**255**

**260**

**29**

**95**

Cytoplasm

Intracellular

Cytosol

Ribosomal submit

Ribosome

Cellular anatomical entity

Intracellular organelle

Organelle

Cytosolic ribosome

Cell junction

**A**

**Supplement Figure S1.** Enrichment analyses of upregulated (A) and downregulated proteins (B) for GO term cellular components in Perm1^-/-^ hearts as compared with WT hearts. The white numbers in bar graphs of the enrichment analysis indicate the number of proteins that were detected in each pathway.

**C**

*

*

WT

Perm1^-/-^

*

*

*

**B**

**A**

Relative mRNA level

Relative mRNA level

Relative mRNA level

**Supplement Figure S2.** qPCR analysis of WT and Perm1^-/-^ hearts (n=5-7 per group). **A,** The gene expression of glucose transporter 1 and 4 (GLUT1 and GLUT4) was increased in Perm1^-/-^ hearts as compared to WT hearts (p=0.01 in GLUT1, p<0.003 in GLUT4), concomitant with the increased levels of glycolytic and polyol pathways (Fig. 4-5). **B,** The mRNA level of CD36, an essential transporter of unesterified fatty acids through the plasma membrane, was significantly downregulated in Perm1^-/-^ hearts. Of note, we observed the reduced myocardial contents of free fatty acids, diglycerides, and triglycerides in Perm1^-/-^ hearts (Fig. 4-5). There was a trend that the expression of CPT1b was reduced in Perm1^-/-^ hearts (FC=0.84), however, the difference was not significant (both p>0.05). There was no change in CPT2 expression in Perm1^-/-^ hearts. (*: p<0.05)


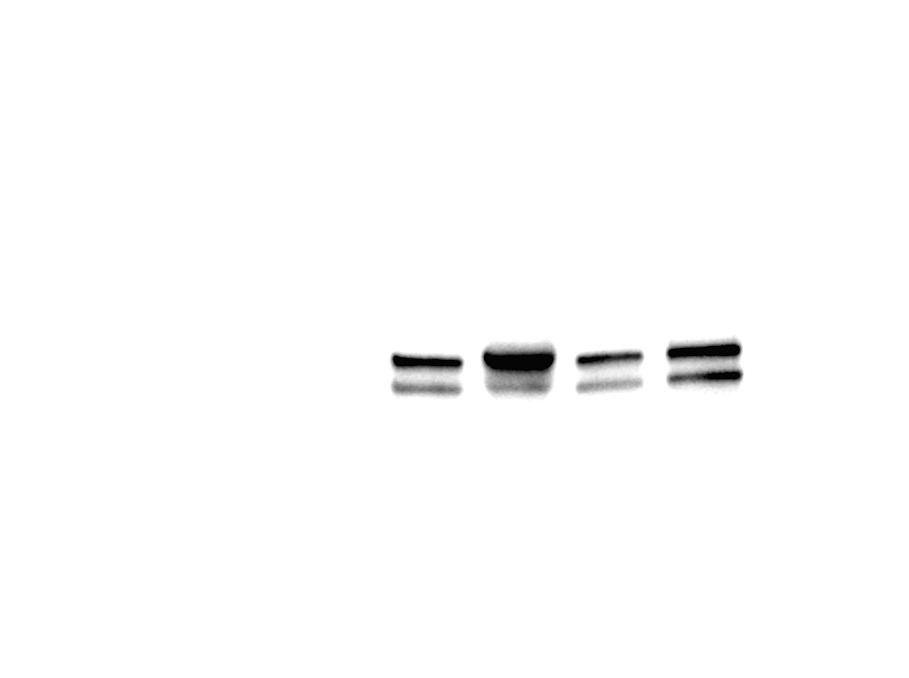


T

N

C

M

Perm1

β-tubulin

(50kDa)

90kDa(b)

100kDa(a)


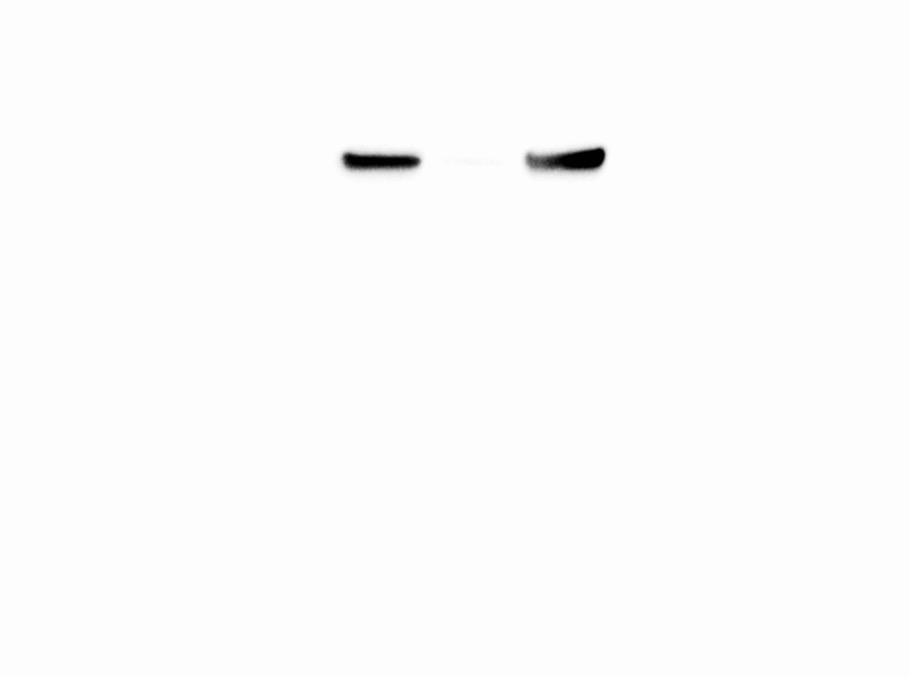

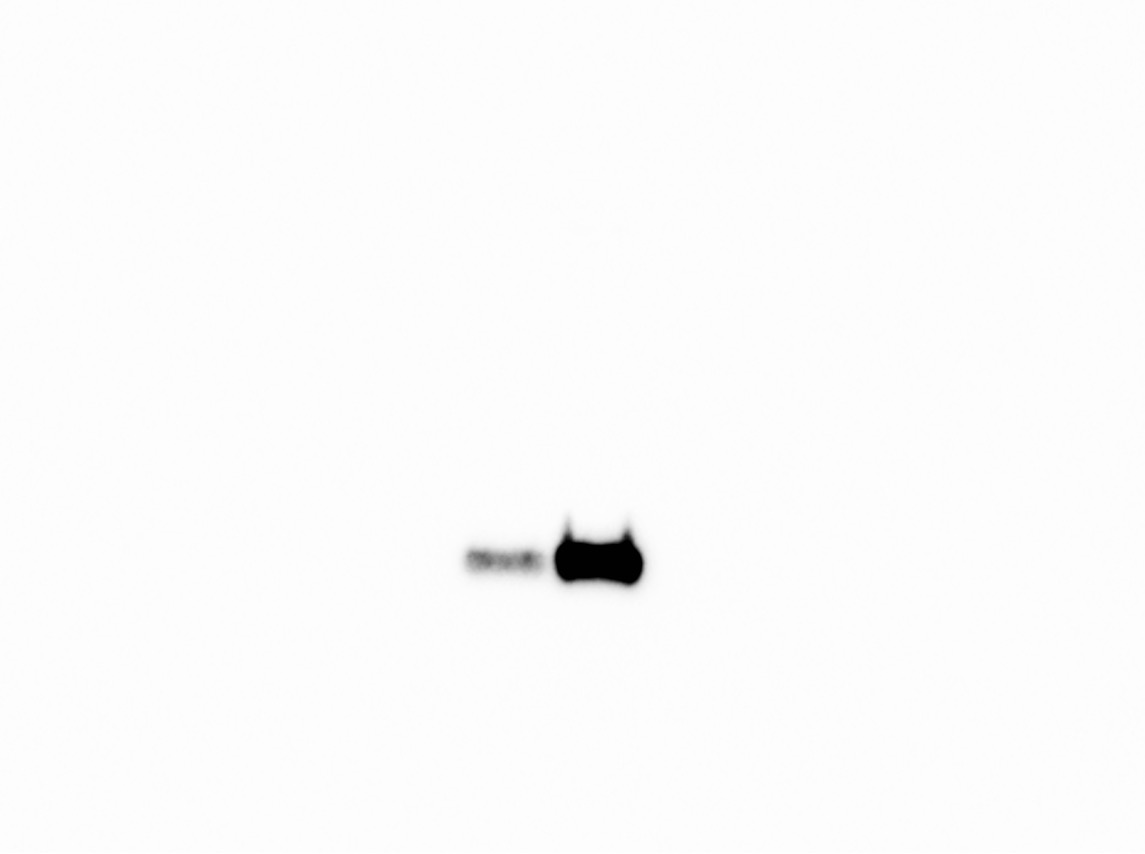


H3

(15kDa)

Hsp60

(kDa)


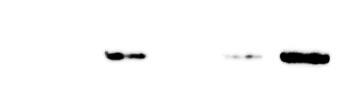


**Supplement Figure S3.** Subcellular fractionation of cardiac tissue from the adult mouse heart (wild-type). Perm1 was detected using the Perm1 antibody, antibodies against cytoplasmic β-tubulin, mitochondrial Hsp60, and nuclear histone H3 (H3) were used to assess the purity of the cytoplasmic (C), mitochondrial (M), and nuclear (N) fractions. Perm1 expression in the whole lysate (total, T) was also included. The nuclear and cytoplasmic fractions as well as the whole lysates were further subjected to co-immunoprecipitation to assess the interaction with ERRα, shown in Figure 5A.

REFERENCE

1. Bhat S, Chin A, Shirakabe A, et al. Recruitment of RNA Polymerase II to Metabolic Gene Promoters Is Inhibited in the Failing Heart Possibly Through PGC-1alpha (Peroxisome Proliferator-Activated Receptor-gamma Coactivator-1alpha) Dysregulation. *Circ Heart Fail*. Mar 2019;12(3):e005529. doi:10.1161/circheartfailure.118.005529

2. Oka SI, Sabry AD, Horiuchi AK, et al. Perm1 regulates cardiac energetics as a downstream target of the histone methyltransferase Smyd1. *PLoS One*. 2020;15(6):e0234913. doi:10.1371/journal.pone.0234913

3. Warren JS, Tracy CM, Miller MR, et al. Histone methyltransferase Smyd1 regulates mitochondrial energetics in the heart. *Proc Natl Acad Sci U S A*. Jul 30 2018;doi:10.1073/pnas.1800680115

4. Shibayama J, Yuzyuk TN, Cox J, et al. Metabolic remodeling in moderate synchronous versus dyssynchronous pacing-induced heart failure: integrated metabolomics and proteomics study. *PLoS One*. 2015;10(3):e0118974. doi:10.1371/journal.pone.0118974

5. Xia J, Sinelnikov IV, Han B, Wishart DS. MetaboAnalyst 3.0--making metabolomics more meaningful. *Nucleic Acids Res*. Jul 1 2015;43(W1):W251-7. doi:10.1093/nar/gkv380

6. Oka SI, Byun J, Huang CY, et al. Nampt Potentiates Antioxidant Defense in Diabetic Cardiomyopathy. *Circ Res*. Jun 25 2021;129(1):114-130. doi:10.1161/circresaha.120.317943

7. Nakamura M, Liu T, Husain S, et al. Glycogen Synthase Kinase-3α Promotes Fatty Acid Uptake and Lipotoxic Cardiomyopathy. *Cell Metab*. May 7 2019;29(5):1119-1134.e12. doi:10.1016/j.cmet.2019.01.005

8. Franceschini A, Szklarczyk D, Frankild S, et al. STRING v9.1: protein-protein interaction networks, with increased coverage and integration. *Nucleic Acids Res*. Jan 2013;41(Database issue):D808-15. doi:10.1093/nar/gks1094

9. Cox J, Mann M. MaxQuant enables high peptide identification rates, individualized p.p.b.-range mass accuracies and proteome-wide protein quantification. Evaluation Studies

Research Support, Non-U.S. Gov't. *Nat Biotechnol*. Dec 2008;26(12):1367-72. doi:10.1038/nbt.1511

10. Luber CA, Cox J, Lauterbach H, et al. Quantitative proteomics reveals subset-specific viral recognition in dendritic cells. Research Support, Non-U.S. Gov't. *Immunity*. Feb 26 2010;32(2):279-89. doi:10.1016/j.immuni.2010.01.013

11. Tong M, Saito T, Zhai P, et al. Alternative Mitophagy Protects the Heart Against Obesity-Associated Cardiomyopathy. *Circ Res*. Dec 3 2021;129(12):1105-1121. doi:10.1161/circresaha.121.319377
